# Altered neural connectivity in excitatory and inhibitory cortical circuits in autism

**DOI:** 10.3389/fnhum.2013.00609

**Published:** 2013-09-27

**Authors:** Basilis Zikopoulos, Helen Barbas

**Affiliations:** Neural Systems Laboratory, Department of Health Sciences, Boston UniversityBoston, MA, USA

**Keywords:** prefrontal cortex (PFC), parvalbumin-positive interneurons, anterior cingulate cortex, ratio of excitation and inhibition, myelinated axons, GAP-43, white matter, short-range and long-distance pathways

## Abstract

Converging evidence from diverse studies suggests that atypical brain connectivity in autism affects in distinct ways short- and long-range cortical pathways, disrupting neural communication and the balance of excitation and inhibition. This hypothesis is based mostly on functional non-invasive studies that show atypical synchronization and connectivity patterns between cortical areas in children and adults with autism. Indirect methods to study the course and integrity of major brain pathways at low resolution show changes in fractional anisotropy (FA) or diffusivity of the white matter in autism. Findings in *post-mortem* brains of adults with autism provide evidence of changes in the fine structure of axons below prefrontal cortices, which communicate over short- or long-range pathways with other cortices and subcortical structures. Here we focus on evidence of cellular and axon features that likely underlie the changes in short- and long-range communication in autism. We review recent findings of changes in the shape, thickness, and volume of brain areas, cytoarchitecture, neuronal morphology, cellular elements, and structural and neurochemical features of individual axons in the white matter, where pathology is evident even in gross images. We relate cellular and molecular features to imaging and genetic studies that highlight a variety of polymorphisms and epigenetic factors that primarily affect neurite growth and synapse formation and function in autism. We report preliminary findings of changes in autism in the ratio of distinct types of inhibitory neurons in prefrontal cortex, known to shape network dynamics and the balance of excitation and inhibition. Finally we present a model that synthesizes diverse findings by relating them to developmental events, with a goal to identify common processes that perturb development in autism and affect neural communication, reflected in altered patterns of attention, social interactions, and language.

## Introduction—the general hypothesis for disrupted connectivity in ASD

The balance of excitation and inhibition is disrupted in autism spectrum disorders (ASD) with widespread repercussions on neural communication (Rubenstein and Merzenich, 2003; Amaral et al., 2008; Rubenstein, 2011). Connections are the conduit for neural communication, forming local or interareal circuits, which collectively construct large scale networks. In the primate brain, cortico-cortical, and cortico-subcortical pathways that travel through the white matter originate from excitatory neurons. The white matter pathways, which consist largely of axons of excitatory neurons, can be subdivided into short/medium- or long-range based on the distance they travel to connect with other neural structures. When these pathways reach their targets in the cortex or in subcortical structures they form excitatory synapses with local excitatory or inhibitory neurons, participating in local microcircuits within a column/minicolumn, or neighboring columns in the cortex, or within subcortical structures. Axons from inhibitory neurons in primates are largely confined within the gray matter and innervate nearby neurons found in the same or different layers within the same or neighboring columns.

This brief description of structural connectivity highlights multiple levels at the macro and micro scales that may be disrupted in varying degrees in ASD, affecting neural communication, and the balance of excitation and inhibition. Converging evidence from genetic, functional, and structural studies suggests that there are changes in excitatory and inhibitory neural communication in ASD and in the structure of the underlying cortical circuits or networks. At the microcircuit and synaptic level, numerous genetic studies have highlighted a large variety of polymorphisms and epigenetic factors that primarily affect neurite growth, synapse formation, and synaptic transmission of excitatory and inhibitory neurons (see Samaco et al., 2005; Hogart et al., 2007; Weiss et al., 2009; Gilman et al., 2011; Hallmayer et al., 2011; Hussman et al., 2011; Voineagu et al., 2011; Shulha et al., 2012; reviewed in Geschwind, 2011). At the level of the network, most imaging studies have also focused on affected brain systems by identifying abnormalities in the gray and white matter, primarily in frontal and temporal lobes, or in their major pathways (Belmonte et al., 2004a; Herbert, 2005; Casanova, 2007; Courchesne et al., 2007; Kumar et al., 2009; Schumann et al., 2010; Schipul et al., 2011; Just et al., 2012).

However, there is a paucity of data about specific changes in neural elements that form excitatory and inhibitory brain circuits and underlie mechanisms of imbalance in ASD. While in short supply, studies at the cellular level have described changes in the cytoarchitecture, density and neurochemical features of excitatory and inhibitory neurons in frontal and temporal areas in autism (Bauman and Kemper, 2005; Casanova, 2007; Amaral et al., 2008; Schmitz and Rezaie, 2008; Blatt and Fatemi, 2011; Penzes et al., 2011; Schumann and Nordahl, 2011; Srivastava et al., 2012). Only a few studies have employed a combination of high resolution methods to study the neural pathophysiology of autism, by identifying specific structural, neurochemical, and molecular changes of neuronal elements that may underlie atypical development of synaptic interactions within functional cortical networks (Weidenheim et al., 2001; Garbern et al., 2010; Zikopoulos and Barbas, 2010). The present review focuses on these structural aspects that likely tip the balance of excitation and inhibition at the level of circuits and networks in ASD.

Several cortical and subcortical areas including frontal and temporal cortices, the amygdala, and the cerebellum exhibit atypical functional and structural characteristics in ASD; it should be noted however, that pathology may also be present in other and as yet not studied brain regions. Frontal cortical pathways have received considerable attention because they consistently show functional disruption in ASD (Hill, 2004; Pickett and London, 2005; Wass, 2011; Just et al., 2012). For this reason, here we focus on three robustly interconnected prefrontal regions: anterior paracingulate and cingulate areas (referred thereafter as ACC) in the medial prefrontal cortex, orbitofrontal cortex (OFC) in the ventral and ventrolateral prefrontal cortex, and lateral prefrontal areas (LPFC). These areas have a key role in attention, social interactions, emotions, and executive control (Barbas, 2000a,b; Barbas et al., 2011), in processes that are severely affected in autism (Baron-Cohen, 1991; Ozonoff et al., 1991; Carper et al., 2002; Maestro et al., 2002; Sparks et al., 2002; Mundy, 2003; Hill, 2004; Girgis et al., 2007; Jiao et al., 2010). In some cases we include relevant findings in temporal or parietal cortices that are connected with the above prefrontal cortices.

The ACC, OFC, LPFC and their pathways are functionally disorganized in autism. There is evidence that at least some of these areas exhibit local over-connectivity and long-distance disconnection (Casanova et al., 2002b; Barnea-Goraly et al., 2004; Casanova, 2004; Herbert et al., 2004; Courchesne and Pierce, 2005; Herbert, 2005; Kana et al., 2006b; Girgis et al., 2007; Just et al., 2007; Pardini et al., 2009; Assaf et al., 2010; Hyde et al., 2010; Anagnostou and Taylor, 2011; Bernardi et al., 2011; Wass, 2011). Aberrant function of ACC in autism includes hyperactivity during response monitoring and social target detection (Thakkar et al., 2008; Dichter et al., 2009) and desynchronized activity during working memory tasks (Kana et al., 2006b), while LPFC shows lower activity in working memory tasks (Luna et al., 2002; Koshino et al., 2008; reviewed in Schipul et al., 2011). Activity in LPFC and OFC is correlated with intellectual level and predicts poor performance of individuals with autism in neuropsychological tasks (Loveland et al., 2008). In addition, in autism there is decreased functional connectivity between OFC, other areas that process emotions, reward, and social interactions, like the amygdala or insula, and language areas in the posterior superior temporal sulcus (Sabbagh, 2004; Bachevalier and Loveland, 2006; Hardan et al., 2006; Girgis et al., 2007; Abrams et al., 2013).

The goal of this article is to synthesize recent high resolution neuropathological findings at the cellular level of circuits and relate the observed changes to relevant gross anatomical, functional, genetic, or epidemiological data. The focus is on axons and neurons that form local or distant circuits. We highlight similarities and differences in the way local vs. long-distance circuits may be affected in ASD and propose refinements to the hypothesis of disrupted connectivity in ASD that may reconcile conflicting findings regarding the prevalence and significance of over-connectivity or under-connectivity in frontal and temporal networks. We additionally report preliminary findings of changes in the ratio of distinct types of inhibitory neurons in dorsolateral prefrontal area 9 of adults with ASD. This pilot study presents novel evidence that addresses the overarching hypothesis of disruption in the balance of excitation and inhibition in autism. Finally, by grounding findings within a developmental framework we propose potential common mechanisms that may underlie the disruption of neural communication and the imbalance of excitation and inhibition in ASD.

## What brings about changes in structural connectivity?

Structural connectivity can change by direct alterations in the physical connections between neurons, reflected in the numbers of synapses, and the biophysical attributes of individual synapses that affect synaptic efficacy. Significant structural changes likely affect functional connectivity, reflected in ASD as atypical synchronization and connectivity patterns of frontal or temporal areas in children and adults with autism, suggesting abnormal engagement and interactions of short-range and long-range excitatory pathways and local inhibitory circuits (Rubenstein and Merzenich, 2003). The study of structural connectivity at the synaptic level in humans is challenging, primarily due to limited tissue availability and variability in tissue preservation that may impede rigorous analyses. Despite these limitations there is considerable evidence for changes in neuronal elements in cortical areas that could affect synaptic function in ASD. Studies report changes in the structure of presynaptic and post-synaptic elements, pathways in the white and gray matter, and density and size of various neuronal and glial cell types, as elaborated below.

## Axon pathology is at the core of atypical connectivity in ASD

Imaging studies in children and adults with autism, show decreased functional connectivity between frontal and other areas and gross changes in the structural integrity of frontal gray and white matter (Barnea-Goraly et al., 2004; Kana et al., 2006a; Just et al., 2007; Keller et al., 2007; Minshew and Williams, 2007; Koshino et al., 2008; Thakkar et al., 2008; Pardini et al., 2009; Minshew and Keller, 2010). Typical findings in the white matter include lower fractional anisotropy (FA) and higher radial diffusivity in ASD groups than in controls, which may come about by a reduction of diffusion barriers between axons (reviewed in Muller et al., 2011). These findings suggest decreased axon diameter and/or decreased myelination that reduce axon volume, and may result in changes in the density of axons.

**Figure 1:**
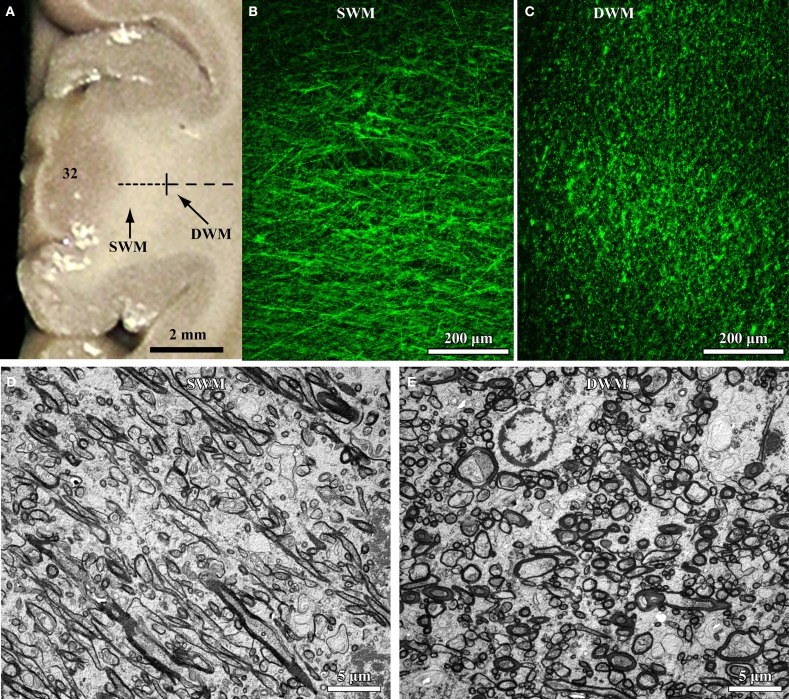
**High resolution segmentation of the white matter. (A)** Coronal view of a representative ACC (A32) tissue slab. Dotted lines indicate gross (macroscopic) distinction of superficial (SWM) and deep (DWM) white matter, based on subsequent microscopic analysis. **(B,C)** Fluorescent photomicrographs of coronal sections from A32 white matter after labeling of axons with a neurofilament protein antibody (NFP-200; green). Light microscopic segmentation of superficial **(B)** and deep **(C)** white matter is based on the distinct orientation of axons at different depths from the gray matter. Axons in the superficial white matter travel mainly perpendicular to the surface of the cortex (**B**, axons appear mainly as thin lines), whereas in the deep white matter most axons travel parallel to the cortical surface (**C**, axons appear mainly as green dots). **(D,E)** EM photomicrographs show mostly elongated axon profiles in the superficial white matter **(D)** and mostly circular axon profiles in the deep white matter **(E)**. Adapted from Zikopoulos and Barbas (2010).

The relative position and size of axons in the white matter below the cortex can be used as an indicator of their termination in nearby or distant brain areas. The deep (inner or sagittal) white matter includes long-range excitatory pathways (Herbert et al., 2004; Hilgetag and Barbas, 2006; Petrides and Pandya, 2006, 2007; Schmahmann and Pandya, 2006; Sundaram et al., 2008), with thicker axons than found in the superficial white matter just below the cortex (Zikopoulos and Barbas, 2010; Figure 1). The superficial (outer or radiate) white matter is situated below cortical layer 6, and carries mostly thin excitatory fibers as axons branch to connect with nearby areas (Figure 1).

Based on the relationship of pathways within the white matter, functional imaging and physiological studies have shown that long-range cortico-cortical pathways that link frontal areas with other cortices are weak and disorganized in autism. Specifically, there is reduced coherence and correlation in task-related activity of distant areas, which constitutes decreased functional connectivity (Just et al., 2004, 2007; Courchesne and Pierce, 2005). In addition, gross structural imaging studies have shown reduced size, FA, and diffusivity in deep white matter tracts, suggesting differential composition or compromised structural integrity of long-distance pathways in adults and children with autism (Alexander et al., 2007; Just et al., 2007; Frazier and Hardan, 2009; Casanova et al., 2011; Jou et al., 2011; Shukla et al., 2011a). In contrast, gross structural imaging studies have reported transient enlargement of the superficial white matter in the frontal cortex of children with autism (Belmonte et al., 2004a; Herbert et al., 2004; Herbert, 2005). Concomitantly, functional studies have shown aberrant or excessive activation and increased synchrony within frontal cortices, suggesting local overconnectivity in autism (Courchesne and Pierce, 2005; Kennedy et al., 2006).

**Figure 2:**
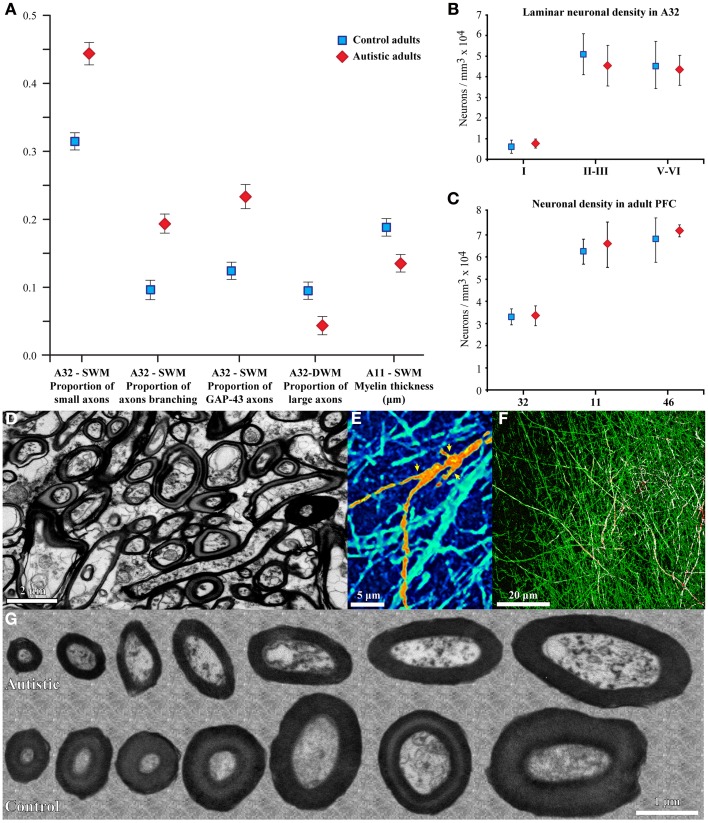
**Changes in myelinated axons below prefrontal cortices in adults with ASD. (A)** In the superficial white matter (SWM) below ACC (area 32) the relative density of small (thin) axons (±SEM) is increased in the autistic cases, and more axons branch and express GAP-43. These data suggest increased local connectivity of ACC in ASD. In contrast, in the deep white matter (DWM) below ACC the relative density of large axons is reduced in ASD, suggesting weakening of long-range connectivity. Thinning of the myelin in axons of all sizes just below OFC (area 11) suggests weak local connections. **(B,C)** Laminar and overall neuronal density below ACC, OFC, and LPFC is similar in adults with ASD and controls and is not correlated with the changes in axons below PFC. **(D)** EM photomicrograph of axons in the superficial white matter below ACC of an adult with ASD. **(E)** Collapsed image (z-projection) from a three-dimensional confocal stack shows myelinated axons branching, labeled with NFP-200 (green). A branching axon is pseudo colored with orange/yellow hue for visualization (yellow arrowheads point to branches). **(F)** Image from a three-dimensional confocal stack with double immunofluorescence shows GAP-43 (red) in axons labeled with NFP-200 (green). Some myelinated axons contain GAP-43 in their axolemma, which is transported to axon terminals and branching points. Colocalization of the two antibodies is rendered white. **(G)** EM photomicrographs show differences in myelin thickness in OFC between control and autistic adults, apparent in all axon size groups.

Our recent work in adult human *post-mortem* brain tissue (Zikopoulos and Barbas, 2010) provides novel evidence for specific structural and molecular changes in individual prefrontal axons (Figure 2). In agreement with the long-range underconnectivity hypothesis, we found that below the anterior cingulate/paracingulate cortices (ACC) in the brains of adults with autism there are fewer large myelinated axons in the deep white matter, which link distant areas (Herbert et al., 2004; Hilgetag and Barbas, 2006; Petrides and Pandya, 2006, 2007; Schmahmann and Pandya, 2006; Sundaram et al., 2008). In sharp contrast, we found a higher density of thin myelinated axons in the superficial white matter below ACC, which was partially due to excessive branching of thin and medium-sized axons, which link nearby areas. In addition, axons below OFC had thinner myelin in ASD cases than in controls (Figure 2). The thinner myelin in OFC was not due to a reduction in the density of oligodendroglia in the white matter (Zikopoulos and Barbas, 2010).

The significance of these findings is twofold. First, the ACC has a key role in attentional control (Gehring and Knight, 2000; Paus, 2001; Ito et al., 2003; Johnston et al., 2007), and OFC in emotions (Barbas and Zikopoulos, 2006; Zikopoulos and Barbas, 2012), and both processes are seriously disrupted in autism (Gomot et al., 2006; Steele et al., 2007; Vlamings et al., 2008; Norbury et al., 2009; Markram and Markram, 2010; Bernardi et al., 2011). Second, in non-human primates, the ACC has the most widespread connections with other prefrontal cortices (Barbas et al., 1999). The OFC is distinguished for its multimodal input from every sensory modality through high-order sensory association and multimodal cortices (Barbas, 1993; Barbas and Zikopoulos, 2006). These findings suggest that changes in axons below ACC and OFC have widespread repercussions on prefrontal networks and beyond. That is why, even though axon features below lateral prefrontal cortices (LPFC) appear unaffected (Zikopoulos and Barbas, 2010), the altered white matter composition below ACC and OFC changes the relationship among prefrontal areas. The changes in the relationship of axons below prefrontal areas could affect LPFC function, because these regions are robustly interconnected in primates (Petrides and Pandya, 1988; Seltzer and Pandya, 1989; Barbas et al., 1999; Barbas, 2000a; Fullerton and Pandya, 2007; Schmahmann et al., 2007).

Two well-studied networks can be used to illustrate additional, and perhaps more specific, implications for the pathology of intrinsic or distant prefrontal circuits in ASD. First, studies of the ACC-LPFC intrinsic circuit in non-human primates show that ACC sends a robust feedback projection that targets primarily the superficial layers of LPFC (Medalla and Barbas, 2009, 2010, 2012). As is typical in cortico-cortical networks in primates, excitatory axons from ACC mainly target excitatory pyramidal neurons in LPFC. However, a smaller but significant proportion (~20%) of excitatory ACC axons form synapses with inhibitory neurons in the superficial layers of LPFC, where they innervate preferentially calbindin (CB) inhibitory neurons (Medalla and Barbas, 2009). Anatomic, physiologic, and computational studies have shown that CB inhibitory neurons innervate the distal dendrites of excitatory pyramidal neurons (Peters and Sethares, 1997) and modulate their activity, increasing the signal-to-noise ratio (Peters and Sethares, 1997; Gonzalez-Albo et al., 2001; Wang et al., 2004). These synaptic specializations suggest that ACC can enhance relevant signals and reduce noise in LPFC, to facilitate focusing attention on a task, and are especially useful during challenging cognitive tasks (Gehring and Knight, 2000; MacDonald et al., 2000; Paus, 2001; Schall, 2001; Ito et al., 2003; Badre and Wagner, 2004; Johnston et al., 2007; Tanji and Hoshi, 2008). The exuberance of thin, short-range axons found in adults with autism (Zikopoulos and Barbas, 2010) that link ACC with nearby areas, including LPFC, suggests a potential exaggeration of this mechanism that could underlie the difficulty of even high-functioning individuals with autism to shift attention. Distant regions that are likely affected are temporal lobe structures, including auditory or multimodal temporal cortices and the amygdala, which have strong bidirectional interactions with prefrontal cortices in non-human primates (e.g., Barbas and Mesulam, 1985; Barbas et al., 1999, 2005b; Ghashghaei and Barbas, 2002; Germuska et al., 2006; Ghashghaei et al., 2007; Medalla et al., 2007; Zikopoulos et al., 2008).

In spite of the small number of cases and heterogeneity on the ASD spectrum, changes in axons below ACC were present in all autistic cases studied, suggesting a fundamental autism phenotype in axons below some prefrontal areas (Zikopoulos and Barbas, 2010). The power and generalizability of these findings are high likely because the cases were well-matched and within a narrow age range (30–44 years), obviating differences in the developmental trajectory that can increase variability. Importantly, the findings are based on multiple independent methods to estimate the same or related variables. For example, axon size and branching were independently evaluated both at the confocal and electron microscopes, and additionally corroborated by independently labeling and estimating the proportion of axons that express axon growth factors, as elaborated below.

### Molecular mechanisms that regulate axon growth are affected in autism

In the study of adults with autism (Zikopoulos and Barbas, 2010), supernumerary branching, and density of thin axons below ACC are associated with increased expression of the Growth Associated Protein 43 (GAP-43; Figure 2). This intracellular protein is produced in the cell body and is quickly transported down the axon to reach branching points, growth cones, and axon terminals (reviewed in Benowitz and Routtenberg, 1997). It is, therefore, most abundant in the superficial part of the white matter and in the gray matter, as axons branch to innervate their targets. GAP-43 also promotes neurotransmitter release, endocytosis and synaptic vesicle recycling (Denny, 2006). These actions are contingent upon phosphorylation of GAP-43 by protein kinase C, which induces local actin filament-membrane attachment. GAP-43 is expressed at high levels during late prenatal and early postnatal stages of axon growth, and is subsequently markedly reduced with the onset of myelination (Kapfhammer and Schwab, 1994; Benowitz and Routtenberg, 1997). In the adult brain GAP-43 is found in significant amounts only in some limbic areas, including the hippocampus and ACC, where it also promotes axon growth, and acts as a lateral stabilizer of actin filaments presynaptically, strengthening synapses to promote long-term potentiation, spatial memory formation, and learning (Maviel et al., 2004; Holahan et al., 2007; Holahan and Routtenberg, 2008). In addition, GAP-43 is found at focal sites after brain injury, where it promotes axon sprouting and regeneration (Neve et al., 1988; Benowitz and Routtenberg, 1997).

In autism, an increase in GAP-43 may persist in adulthood in response to reported inflammation (Vargas et al., 2005; Morgan et al., 2010), or due to axon damage. Interestingly, GAP-43 transcription is directly regulated by calcineurin and nuclear factor of activated T cells (Yoshida and Mishina, 2005; Nguyen et al., 2009), which are targets of immunosuppressants like rapamycin (Ho et al., 1996). Rapamycin inhibits the mTOR signaling pathway, and improves neurological dysfunction in animal models of tuberous sclerosis that are relevant for autism (Ehninger and Silva, 2011). Therefore, it seems plausible that GAP-43 and related signaling proteins may provide the link between neurological deficits and the extensive immune dysregulation in autism (Smith et al., 2007; Atladottir et al., 2009; Becker and Schultz, 2010; Patterson, 2011; Garbett et al., 2012; Hsiao et al., 2012; Malkova et al., 2012; Patterson, 2012).

A variety of external factors up-regulate GAP-43 expression, including estrogenic agents that disrupt endocrine function, such as bisphenol A, and immunosuppressive and psychiatric drugs used for a variety of common disorders, including psoriasis, asthma, rheumatoid arthritis, dry eye, depression, and anxiety (Wong et al., 1989; Jyonouchi et al., 2001; Granda et al., 2003; Croen et al., 2005, 2011; Ostensen et al., 2006; Sairanen et al., 2007; Brown, 2009; Nguyen et al., 2009). Several of these substances came into heavy use in the early 80s at a time when the prevalence of autism began to rise (Blaxill, 2004). The use of endocrine disruptors during pregnancy has been correlated with increased autism risk (Croen et al., 2011; Simpson et al., 2011; de Cock et al., 2012).

Information on the developmental trajectory of axon growth and relevant signaling pathways will help delineate a more detailed timeline for the development of autism pathology, narrow down the temporal window for the insult, and spur new research to identify affected signaling pathways and factors that may be targeted for therapeutic interventions. Importantly, epidemiologic studies are necessary to investigate the relationship between signaling pathways and possible cumulative effects of environmental agents, diet, and drugs on the uterine and postnatal environment that may perturb the expression of factors implicated in axon growth and guidance in autism.

## Dendritic spine pathology in ASD

Structural evidence for the disturbance of neural communication in ASD is also apparent in the cortical gray matter, specifically on post-synaptic targets of cortical or subcortical afferents, the dendrites of excitatory pyramidal neurons. In dorsolateral prefrontal area 9, temporal area 21, and parietal area 7, there is increased dendritic spine density in layer II pyramidal neurons, and in neurons of layer V only in area 21, among those studied (Hutsler and Zhang, 2010). These differences were found in all major dendritic branches (apical, basilar, and oblique), and along the length of apical dendrites of pyramidal cells for several hundred micrometers from the cell body. Based on these results, ASD seems to be part of a small group of developmental disorders where there is no apparent loss of dendritic spines.

Since the majority of synapses on spines of pyramidal neurons are excitatory (e.g., Lowenstein and Somogyi, 1991; Peters et al., 1991; Ahmed et al., 1997; Somogyi et al., 1998; Alonso-Nanclares et al., 2004; Douglas and Martin, 2004; Anderson and Martin, 2009; Medalla and Barbas, 2009, 2010; Micheva et al., 2010), changes in spine density suggest an alteration in the density of excitatory synapses on dendritic segments within prefrontal, temporal, and parietal cortices in ASD. However, one cannot rule out possible changes in the density of inhibitory synapses onto cortical neurons, which also target dendritic spines and shafts in various ratios, depending on the pathway. Moreover, preliminary morphological analysis (Hutsler and Zhang, 2010; Avino et al., 2012) shows immature morphology and excessive fluctuation in the length and shape of spines in ASD cases, suggesting synaptic lability. The same morphological changes could affect dendritic cytosolic compartmentalization, dendritic computations, and ultimately neuronal processing (for a review see London and Hausser, 2005).

The findings on spine features are limited to studies by one group so far and do not offer explicit clues about the potential local or distant presynaptic origin of the connections affected, but are nevertheless informative about the overall pathology in ASD. Specifically, a consistent finding is increased layer II connectivity in ASD in association areas examined by (Hutsler and Zhang, 2010). Neurons in the superficial layers of the cortex are primarily involved in ipsilateral and contralateral cortico-cortical connections, and receive feedback projections from areas that have fewer layers or lower neuronal density, such as the ACC (Barbas and Rempel-Clower, 1997), and these pathways may be disproportionately affected in ASD. Layer II in LPFC receives strong input from the amygdala (Ghashghaei et al., 2007), most subcortical neuromodulatory systems (Berger et al., 1988; Lewis et al., 1988; Gaspar et al., 1989; Lewis and Morrison, 1989; Raghanti et al., 2008), and the ACC (Barbas et al., 1999; Medalla and Barbas, 2009, 2010). Another type of pathway that targets the superficial cortical layers, including layer II, originates from the widely projecting matrix neurons of the thalamus, which can effectively propagate and synchronize thalamocortical activity over large expansions of the cortex (Zikopoulos and Barbas, 2007; Jones, 2009). It is possible that within the frontal lobe, potential thalamocortical pathology in the upper layers may be restricted to lateral prefrontal areas, because at least the gross features of myelinated thalamocortical axons in the deep white matter below the ACC are not affected in ASD (Zikopoulos and Barbas, 2010). Further work is needed to determine if thalamocortical axons are more specifically affected as they branch to innervate different prefrontal cortices.

Further, based on the inside-out model of development of the cortex, layer II is the last layer to develop. The maturation period of layer II is protracted as connections are formed, in accord with the fact that long-distance cortico-cortical and callosal connections that these superficial layers participate in also develop late. It seems that changes in white matter axons, described in previous sections (Zikopoulos and Barbas, 2010), as well as changes in dendritic spines in the gray matter (Hutsler and Zhang, 2010), point toward late prenatal or early postnatal critical periods for the development of ASD neuropathology. This is also supported by the fact that callosal pathways, which also develop late, are severely compromised in ASD as well (Alexander et al., 2007; Just et al., 2007; Frazier and Hardan, 2009; Jou et al., 2010; Anderson et al., 2011b; Cantlon et al., 2011; Casanova et al., 2011; Fame et al., 2011; Schipul et al., 2011).

The finding of increased dendritic spine density in layer V pyramidal neurons only in temporal area 21 (Hutsler and Zhang, 2010) may be associated with atypical auditory or language processing and with deficits in social-emotional interactions in ASD. This idea is in accord with imaging studies (e.g., Just et al., 2004; Gomot et al., 2006; Bigler et al., 2007; Lee et al., 2007). Within the cortex, atypical activation patterns of layer V neurons in temporal areas may have an effect in feedback pathways to other cortical areas. Moreover, the amygdala, thalamus, and striatum are major subcortical targets of cortical layer V neurons, and structural as well as functional studies indicate that these subcortical structures and their circuits may be affected in autism (e.g., Bauman and Kemper, 1985; Tsatsanis et al., 2003; Schumann et al., 2004; Haznedar et al., 2006; Schumann and Amaral, 2006; Shukla et al., 2010; Tamura et al., 2010; Cheon et al., 2011; Di Martino et al., 2011; Langen et al., 2012).

## Neuronal and glial cell densities and morphology in ASD

Several structural imaging studies have shown that there is abnormal acceleration of brain growth in ASD. The brains of young children with ASD are larger than those of typically developing controls, and although this enlargement is attributed mostly to increased white matter volume, there is also significant enlargement of gray matter, especially in frontal and temporal areas (reviewed in Courchesne et al., 2011a). The white matter or cortical enlargement appears to be transient and is not evident in adults with ASD (Herbert, 2005; Redcay and Courchesne, 2005). In agreement with these data, recent preliminary findings suggest that the increase in gray matter volume in children with ASD may, in some cases, be due to increased number of neurons, at least in some prefrontal cortices (Courchesne et al., 2011b). The authors of this study reported that children with ASD have, on average, 79% more neurons in dorsolateral prefrontal cortices (DLPFCs) and 29% more neurons in mesial prefrontal cortices (mesial: medial prefrontal cortices excluding cingulate areas). An earlier study also reported neuropathological thickening of the subependymal cell layer, multifocal subependymal nodular dysplasia, and heterotopias in some children and adults with ASD (Wegiel et al., 2010). These developmental changes may reflect multiregional cortical and subcortical dysregulation of neurogenesis, neuronal migration, and maturation in ASD.

In the brains of adults with autism there are no significant changes in the overall number or density of neurons (Zikopoulos and Barbas, 2010), or in the laminar density of neurons in medial areas 24, 32, orbital area 11, or dorsolateral areas 9 and 46. This evidence indicates that in autism the numbers of neurons in prefrontal cortices are comparable to controls in adulthood. Several other studies also report no differences in the numbers or density of neurons in other cortical areas, including ventrolateral language-related frontal areas 44 and 45 (Jacot-Descombes et al., 2012), area 23 in the posterior cingulate cortex (PCC) and area 37 in the fusiform gyrus (FFG; Oblak et al., 2011b, but see van Kooten et al., 2008), and in areas 3b, 4, 9, 10, 11, 17, 24, 43, and 44 (Casanova et al., 2002b, 2006) in children or adults with ASD. In line with this evidence, there appear to be no differences in cortical layering and thickness in prefrontal, temporal, and parietal areas of children and adults with ASD (Hutsler et al., 2007; Zikopoulos and Barbas, 2010). However, parts of areas 24 and 23 in the dorsal and posterior cingulate cortices display altered cytoarchitecture with irregularly distributed neurons, leading to irregular lamination and poor demarcation of layers IV and V in some ASD cases (Simms et al., 2009; Oblak et al., 2011b).

Detection of potential changes in the number or density of neurons in ASD additionally depends on the types of neurons analyzed. A recent study showed that children with autism consistently had a significantly higher ratio of von Economo neurons (VENs, also known as spindle neurons) to pyramidal neurons than control subjects in frontoinsular cortex (Santos et al., 2011). The authors of this study posit that higher numbers of VENs in autism may be related to alterations in migration, cortical lamination, and apoptosis, and may also underlie a heightened interoception, described in some clinical observations. It seems though that VEN numbers may be regionally specific and age-dependent, because there are no overall differences between autism and control brains in ACC area 24 in teenagers and young adults (Simms et al., 2009). However, among the autism cases, there were two subsets; 1/3 of the cases had significantly increased VEN density and the remaining 2/3 of the cases had reduced VEN density compared to controls.

Changes in the density of glia in the cortex in ASD appear to be type- and region-specific, as well. In a recent study, we did not find differences in the densities of oligodendrocytes, astrocytes, and microglia in the white matter below OFC (Zikopoulos and Barbas, 2010). However, findings suggest a role of glia in ASD pathology in the gray matter based on increased density of astrocytes in frontal cortices in ASD, although the results were not based on stereological analysis (Cao et al., 2012). Another intriguing finding pertains to a higher density of microglia in the gray matter of DLPFC, accompanied by increased activation of microglia in some ASD cases (Morgan et al., 2010). The same group recently showed that microglia are more frequently present near neurons in DLPFC leading to aberrantly close microglia–neuron association (Morgan et al., 2012). Interestingly, the density of activated microglia is additionally elevated in the gray matter of medial prefrontal, cingulate, orbitofrontal, and the gyral fusiform cortices in ASD (Pardo et al., 2005; Vargas et al., 2005; Suzuki et al., 2013). These findings indicate the potential for neuroinflammation and immune responses in some ASD cases that may be linked to higher levels of GAP-43 (Zikopoulos and Barbas, 2010).

Finally, a frequently observed change in the structure of cortical gray matter in children and adults with ASD is minicolumnopathy, defined by decreased columnar width, characterized by diminished and disrupted peripheral neuropil compartment (Casanova et al., 2002a,b, 2006; Buxhoeveden et al., 2006). More specifically, minicolumns in ASD appear to have less peripheral neuropil space and increased spacing among the constituent cells in several areas (3b, 4, 9, 10, 11, 17, 24, 43, 44). Frontal area 44 seems to be the most affected, and the pathology is evident in children and adults with ASD. The increased number of minicolumns in autism may be accompanied or brought about by changes in the size of neurons, the number of cells per column, or their greater dispersion, resulting in no global difference in neuronal density. In line with this evidence, there are reports of decreased size of pyramidal neurons in layers III, V, VI in language related areas 44, 45 (Jacot-Descombes et al., 2012), in layers I-III and layers V-VI of cingulate area 24b and in cell packing density in layers V-VI of cingulate area 24c (Simms et al., 2009) in children and adults with ASD. In addition, areas 24 and 23 in the ACC and PCC display altered cytoarchitecture and increased density of neurons in the subcortical white matter (Simms et al., 2009; Oblak et al., 2011b). The latter is in agreement with observations of abnormal cell patterning at the cortical gray-white matter border of areas 9, 21, and 7 in ASD (Avino and Hutsler, 2010).

All these reported changes in neuron density and morphology, as well as laminar and columnar distribution, can affect both excitatory and inhibitory connections and circuits. In particular, the peripheral neuropil space surrounding the minicolumn is the conduit for inhibitory and excitatory local circuit projections (Peters and Sethares, 1996; Mountcastle, 1997, 1998; Casanova et al., 2003; Douglas and Martin, 2004) that may also be affected, further tipping the balance of excitation and inhibition in ASD, as elaborated below.

## Structural changes in cortical inhibitory neurotransmission

### Changes in inhibitory neurotransmission in ASD

Key evidence for irregular inhibition patterns in autism comes from functional data, suggesting decreased levels of synchronization during response inhibition tasks (Rubenstein and Merzenich, 2003; Yizhar et al., 2011). In addition, molecular studies of autistic individuals and relevant animal models have identified dysregulation of inhibitory biomarkers and mutations in genes associated with the development of cortical inhibitory neurons and their synaptic communication (Ma et al., 2005; Collins et al., 2006; Selby et al., 2007; Tabuchi et al., 2007; Yip et al., 2008; Fatemi et al., 2009a,b; Chao et al., 2010; Blatt and Fatemi, 2011; Gandal et al., 2012).

Importantly, a number of recent studies have consistently found changes in the levels of GABA receptors in frontal and temporal areas. The mean density of GABA_A_ receptors and the density of benzodiazepine binding sites in all layers of area 24 are decreased in ASD (Oblak et al., 2009). Similar reduction is found in the superficial layers of areas 23 (PCC) and 37 (FFG). In the deep layers of the FFG there is also reduction in the number of benzodiazepine binding sites (Oblak et al., 2011a), found on inhibitory neurons (Murray and Wise, 2012). Interestingly, in the superficial layers of PCC and FFG the autism group appears to have higher binding affinity for ligands of the GABA_A_ receptor. The authors suggest that the observed downregulation of receptors may be the result of increased GABA innervation and/or release. In addition, there are significant reductions in GABA_B_ receptor density in the ACC, PCC and FFG in the brains of people with autism compared to matched controls (Oblak et al., 2010). These changes in the GABA_B_ receptor subtype may contribute to the functional deficits in socio-emotional and cognitive processing, as well as identification of faces and facial expressions by individuals with ASD.

The reduction in GABA receptors and benzodiazepine binding in the cortex is a consistent deficit in autism, with similar findings in the hippocampus (Blatt et al., 2001; Guptill et al., 2007), suggesting widespread GABA receptor abnormalities in ASD. Based on recent findings (Fatemi et al., 2009b) of reduced levels of proteins in three of the GABA_A_ receptor subunits in autism in multiple cortical regions, it is possible that a defect in one or more of the GABA_A_ receptor subunits exists as well. Moreover, genetic studies found significant association and molecular interactions of specific GABA receptor subunit genes in autism (Ma et al., 2005). However, despite the evidence for widespread disruption of inhibitory neurotransmission in the cortex little is known about the state of the GABAergic interneurons themselves in the cortex in ASD (Lawrence et al., 2010; Oblak et al., 2011b), whose organization and function is highlighted below.

### Circuit basis for the initiation of inhibitory control

In the cortex, inhibitory control is primarily mediated through local GABAergic interneurons, which comprise a diverse group distinguished by morphology, the types of neurons and sites they synapse with, physiologic properties, and efficacy of inhibitory control (White, 1989; Kawaguchi and Kubota, 1997; Thomson and Deuchars, 1997; Somogyi et al., 1998; Gupta et al., 2000). Inhibitory neurons represent 20–30% of all neurons in the mammalian neocortex and in the frontal cortex of humans they make up ~21% of the neuronal population (Hornung and De Tribolet, 1994; Kalus and Senitz, 1996; Benes et al., 2001; Sherwood et al., 2010). In primates, inhibitory neurons can be classified by their expression of the calcium-binding proteins parvalbumin (PV), calbindin (CB), and calretinin (CR), which comprise largely non-overlapping neurochemical groups of inhibitory neurons in the cortex (Hendry et al., 1989; Defelipe, 1997). PV labels basket and chandelier inhibitory neurons (Defelipe et al., 1989b; Kawaguchi and Kubota, 1997), which are most prevalent in the middle layers of the cortex, where they form perisomatic synapses on pyramidal neurons, providing strong inhibition (Defelipe et al., 1989b; Shao and Burkhalter, 1999). CB labels several cortical morphologic types of inhibitory neurons, which are most densely distributed in cortical layers 2 and upper layer 3, and innervate distal dendrites of pyramidal neurons (Peters and Sethares, 1997), modulating their activity. CR inhibitory neurons are found mostly in the upper layers (I-IIIa) as well, where they innervate mostly other GABAergic neurons, at least in the upper layers (Gabbott et al., 1997; Meskenaite, 1997; Defelipe et al., 1999; Gonchar and Burkhalter, 1999). This regularity in the laminar distribution of PV, CB, and CR neurons is seen in frontal, temporal, and sensory association areas, which have been studied in primates (Defelipe et al., 1989a, 1990; Conde et al., 1994; Kondo et al., 1999; Dombrowski et al., 2001; Barbas et al., 2005b).

In the cortex there is also regularity in the laminar origin and termination of excitatory pathways, which can be predicted based on the structure of interconnected areas, as described by the structural model for connections (Barbas, 1986; Barbas and Rempel-Clower, 1997; Rempel-Clower and Barbas, 2000). Briefly, according to this model, limbic areas, which have fewer than 6 layers and lower cell density, send mainly feedback projections to eulaminate areas, which have 6 layers and higher cell density. These projections originate mainly from the deep layers and terminate mostly in the superficial layers. Projections in the opposite direction are feedforward, predominantly originate from the superficial layers of eulaminate areas and terminate in the middle/deep layers of limbic cortices. Connections between areas with similar architecture originate and terminate equally in all layers. Numerous studies, have consistently supported this model for ipsilateral and callosal connections among diverse cortices in non-human primates (Barbas, 1986; Barbas and Rempel-Clower, 1997; Barbas et al., 2005a,b; Medalla and Barbas, 2006; Medalla et al., 2007; Bunce and Barbas, 2011), and in other species (Grant and Hilgetag, 2005; Hilgetag and Grant, 2010).

Moreover, a series of studies in non-human primates has established that whereas excitatory prefrontal pathways innervate mostly excitatory neurons at the site of termination, they also innervate a smaller but significant (~10–30%) proportion of inhibitory neurons (Barbas et al., 2005b; Medalla et al., 2007; Medalla and Barbas, 2009, 2010; Anderson et al., 2011a; Bunce and Barbas, 2011; reviewed in Barbas and Zikopoulos, 2007). These findings provide the circuit basis for initiation of inhibitory control by prefrontal areas. Connections thus originate and terminate in distinct laminar microenvironments where the distribution of specific classes of inhibitory neurons also varies, providing the framework to examine the structural underpinnings for the imbalance in excitation and inhibition in autism, as elaborated in the preliminary experiments presented below.

### Decreased ratio of PV/CB inhibitory neurons in dorsolateral prefrontal area 9 in ASD

**Figure 3:**
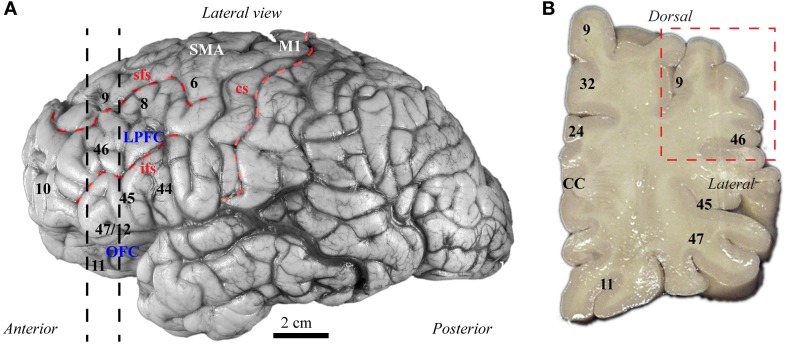
**Map of human frontal areas. (A)** Lateral view of the human brain shows the dorsolateral prefrontal area 9 and its relationship with other frontal areas. Dotted lines indicate the coronal level used for analysis. **(B)** One centimeter thick slab of frontal cortex shows the region sampled in the dorsolateral prefrontal cortex (red dotted-line square). See Appendix for abbreviations.

The balance of excitation and inhibition is affected in autism with detrimental effects on neural communication. Elements of inhibitory neurons are affected in autism, but the state of distinct neurochemical classes of inhibitory neurons in prefrontal cortex is unknown. Here we performed a preliminary study to examine the laminar distribution of cortical inhibitory neurons in ASD, using *post-mortem* adult human brain tissue from dorsolateral prefrontal area 9 (*n* = 2 autistic; *n* = 2 matched controls for age, sex, and hemisphere; Figure 3; Table 1). We compared the density of two non-overlapping, functionally distinct classes of local inhibitory interneurons, which, in primates, are also neurochemically distinct, based on their expression of the calcium-binding proteins calbindin (CB) or parvalbumin (PV).

**Table 1:** **Clinical characteristics of post-mortem cases studied**.

**Case number**	**Control-4786**	**Control-4981**	**Autism-4541**	**Autism-6677**
Age at death (years)	36	42	44	30
Sex	Male	Male	Male	Male
*Post-mortem* interval (hours)	20	18	31	16
Primary cause of death	Myocardial infarction	Myocardial infarction	Acute myocardial infarction	Congestive heart failure
Hemisphere	Right	Right	Right	Right
**AUTISM DIAGNOSTIC INTERVIEW—REVISED (ADI-R) SCORES FOR AUTISTIC CASES**				
(A) Qualitative impairments in reciprocal social interactions (cutoff: 10)			26	26
(B) Qualitative abnormalities in communication (Verbal; cutoff: 8)			18	22
(C) Qualitative abnormalities in communication (Non-verbal; cutoff: 7)			13	^#^
(D) Restricted, repeated, and stereotyped patterns of behavior (cutoff: 3)			6	12
(E) Abnormality of development evident at or before 36 months (cutoff: 1)			5	5

# Scores were not obtained/not applicable due to lack of communication skills. All donors in the autism group had difficulties with communication, social behaviors, and atypical interests, consistent with a diagnosis of autism, and the ADI-R scores met and exceeded cutoffs for autism in each of these areas.

**Figure 4:**
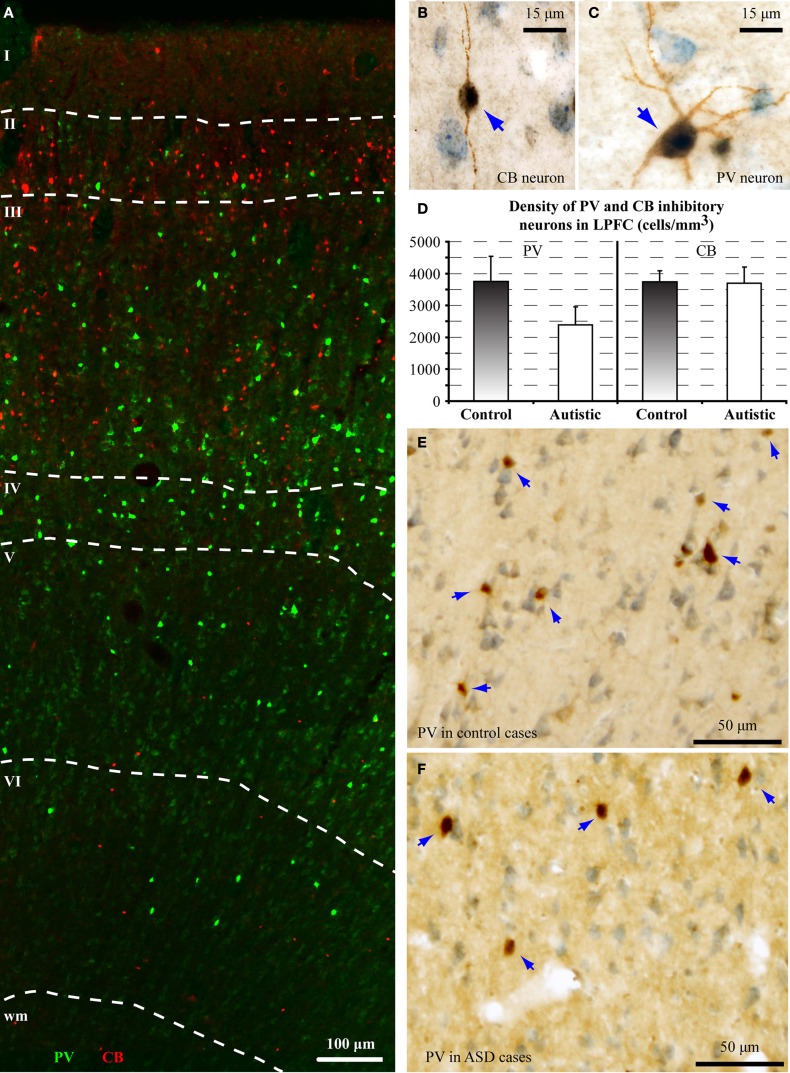
**There is a decrease in the ratio of parvalbumin (PV) to calbindin (CB) inhibitory neurons in area 9 of the human brain in autism. (A)** Fluorescent photomicrograph shows the preferential laminar distribution of CB (red) in the superficial layers and PV (green) in the middle-deep layers of the human dorsolateral prefrontal cortex. **(B,C)** High magnification photographs of CB and PV neurons in the human dorsolateral prefrontal cortex (indicated by blue arrows). **(D)** Preliminary results show lower density of PV neurons in autistic cases (cells/mm^3^ ± standard deviation). **(E,F)** Low magnification photographs of PV neurons in the dorsolateral prefrontal cortex (indicated by blue arrows) of control and ASD adults.

There was a significant reduction of PV neurons in the autistic brains, in both cases [(density: cells/mm^3^ ± standard deviation) control, PV: 3747 ± 786; CB: 3747 ± 337; ASD, PV: 2390 ± 564; CB: 3693 ± 511; *p* = 0.01; Figure 4]. The ratio of PV/CB inhibitory neurons thus decreased by approximately a third in ASD (to 0.65), potentially affecting inhibitory efficacy and overall network dynamics. In typical controls the ratio is close to 1, as is also found in non-human primates (Gabbott and Bacon, 1996; Dombrowski et al., 2001).

PV inhibitory neurons are most prevalent in the middle cortical layers, and provide strong perisomatic inhibition of excitatory neurons (Defelipe et al., 1989b; Kawaguchi and Kubota, 1997; Shao and Burkhalter, 1999). Reduction in PV inhibitory neurons in area 9 may help explain abnormally high columnar activation and desynchronization of oscillatory activity in autism (reviewed in Defelipe, 1999). Our findings are in accord with evidence of compromised inhibitory neurotransmission in autism, reflected by reduced gamma band power of auditory responses in children and adolescents with autism (Wilson et al., 2007), and absence of stimulus-driven synchronization effects on sensory perception (Tommerdahl et al., 2008). These findings suggest atypical coordination of local excitatory-inhibitory cortical activity. Our preliminary findings are also in line with a recent report, showing that in the fusiform face area (FFA) there is less synchrony between alpha and gamma waves, when subjects with autism look at faces, when compared to controls (Khan et al., 2013). Because both of these brain rhythms depend on local inhibition driven primarily by PV neurons (Chow et al., 1998; White et al., 2000; Whittington et al., 2000, 2011; Borgers and Kopell, 2003; Buzsaki and Draguhn, 2004), reduction in phase-amplitude coupling between slow alpha and fast gamma rhythms suggests compromised inhibitory neurotransmission.

On the other hand, we found no differences in the density of CB inhibitory neurons in area 9, which are most numerous in the superficial cortical layers, and have modulatory effects (e.g., Peters and Sethares, 1997; Gonzalez-Albo et al., 2001). CB neurons in LPFC have a role in gain modulation during attentional processes, and among inhibitory classes, they are targeted preferentially by ACC pathways (Medalla and Barbas, 2009).

Previous findings of changes in the white matter suggest that pathways linking ACC with nearby prefrontal areas are excessively dense in autism (Zikopoulos and Barbas, 2010). These findings are consistent with functional studies showing that ACC in autism is hyperactive, especially during response monitoring (Thakkar et al., 2008). This could lead to over activation of CB inhibitory neurons in area 9. This circuit mechanism suggests heightened ability to focus attention, which, on one hand, can be advantageous for complex problem solving. On the other hand, excessive strength in the pathway from ACC to LPFC may also disrupt the ability to shift attention flexibly, and may contribute to the rigid and repetitive behavior seen in autism. In line with this hypothesis, the reported increase in the density of dendritic spines on layer II pyramidal neurons of dorsolateral area 9 (Hutsler and Zhang, 2010), may reflect a plasticity change that spines can undergo (Nimchinsky et al., 2002), perhaps to accommodate the excess fiber input of feedback pathways from ACC in ASD.

A potential change in the ratio of the functionally distinct classes of inhibitory neurons in lateral area 9 in autism can have an impact on the activity of other areas both locally and in widespread distributed circuits, affecting neural dynamics of communication in the cortex. In accordance with our preliminary data, a reduction in PV inhibitory neurons, which mediate perisomatic inhibition of pyramidal excitatory neurons, may diminish strong inhibition in prefrontal areas, leading to over excitation and desynchronization of neuronal activity over large brain networks. This outcome could offer clues on the high prevalence of epilepsy in autism (about 30%) (reviewed in Levisohn, 2007; Hughes, 2008), and has profound implications for LPFC function, like working memory, as reported for autism (Luna et al., 2002; Steele et al., 2007). The ability of LPFC to dynamically adjust the attentional gain in these processes relies heavily on the activity of local PV inhibitory neurons, which underlie shifts in cortical rhythms during cognitive tasks (Abbott and Chance, 2005; Borgers et al., 2008), a process that is also necessary to shift attention flexibly.

To date, DLPFC is the only cortical area in which changes in the ratio of inhibitory neurons in ASD have been reported, since Oblak et al. (2011b) found no differences in parvalbumin, or calbindin interneurons in areas in the posterior cingulate and FFG. It should be noted however, that given the extensive physiological evidence for atypical inhibitory activity patterns in ASD more cortical areas need to be examined. If supported with data from more cases, our findings will have important implications for the pathology in autism. In addition, studies in a variety of animals and humans have established that CB neurons develop earlier than PV neurons (Alcantara et al., 1993; Yan et al., 1997; Letinic and Kostovic, 1998; Hof et al., 1999), and the selective reduction of PV neurons in area 9 in autism suggests the likely timing of the pathology. The status of axons below prefrontal areas also point to changes that have their root in development, as discussed below in the context of a model that relates pathological findings to developmental events.

## A model for the development of disrupted frontal networks in ASD

### Local overconnectivity, long-distance disconnection, or both? it depends on the area

Findings from a variety of functional and structural imaging studies suggest that the breakdown in neural communication in autism involves local overconnectivity and long-distance disconnection, especially in pathways that include the frontal lobe (Herbert et al., 2003; Belmonte et al., 2004b; Carper and Courchesne, 2005; Courchesne and Pierce, 2005; Kennedy et al., 2006; Thakkar et al., 2008). There is general agreement that long-distance connections are weak in autism, but some studies suggest that local connections are also weak, or at least not excessive (e.g., Sundaram et al., 2008; Shukla et al., 2011a,b). The disparity in findings on first blush may be attributed to methodological issues inherent in the limited resolution of MRI and DTI, specific methodological and data analysis choices (reviewed in Muller et al., 2011), or poor contrast of the gray-white matter boundary in autism that renders automatic segmentation ambiguous (Bailey et al., 1998; Avino and Hutsler, 2010).

**Figure 5:**
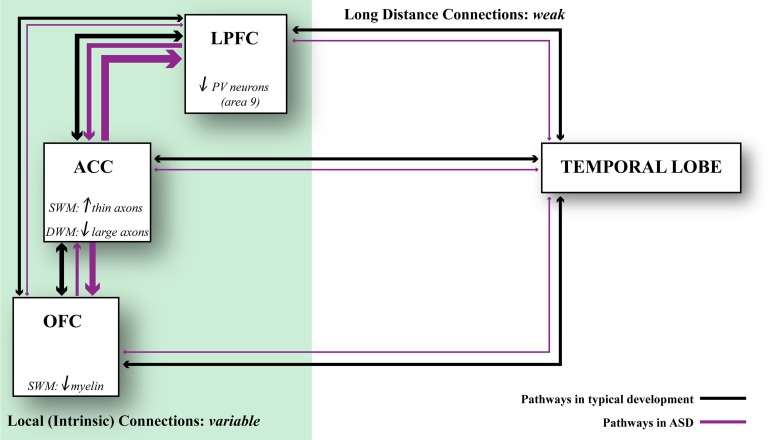
**Relationship of axonal features to developmental events**. Changes in axons and inhibitory neurotransmission affect network dynamics in ASD. ACC exhibits local overconnectivity in ASD, which combined with changes in the ratio of inhibitory neurons in LPFC can tip the balance of excitation and inhibition. OFC exhibits weak local connectivity in ASD due to thinning of the myelin, which may affect conduction velocity. Overall, prefrontal areas exhibit weakening in their long-distance connections. This connectivity pattern is supported by structural and functional data. Black lines indicate typical connectivity and purple lines indicate connectivity in ASD. The thickness of the line indicates the strength of a connection.

The most likely scenario, however, is that connectivity is affected differentially in distinct cortical regions in autism (Figure 5). This hypothesis is consistent with findings that suggest weak local connectivity in some sensory areas or the face region (Sundaram et al., 2008; Shukla et al., 2011a,b; Khan et al., 2013), contrasted with excessive connectivity between some prefrontal cortices in autism (Herbert et al., 2003; Kennedy et al., 2006; Thakkar et al., 2008; Zikopoulos and Barbas, 2010). We found evidence suggesting overconnectivity by the ACC, no change in lateral prefrontal, and weak connectivity in OFC in autism. These findings are based on high resolution methods to view individual axons at the level of the system and to zero in at axon segments at the electron microscope in *post-mortem* brain tissue (Zikopoulos and Barbas, 2010). The high resolution methods employed make it possible to differentiate not only the gray-white matter border, but also to separate the superficial from the deep white matter based on axon orientation. In coronal sections, axons that course in the superficial white matter appear as elongated rods of variable size and direction. In contrast, axons that dive down to the deep white matter en route to distant areas appear as small circular, doughnut-like, structures, because they travel parallel to the cortical surface (Figure 1).

Precise segmentation of the superficial white matter revealed an excess number of medium and thin axons and more branching just below the ACC in the brains of adults with autism (Zikopoulos and Barbas, 2010). The affected superficial white matter links nearby areas. We found no such changes in axons below lateral areas 9, 46, or orbital area 11. But just below area 11 the myelin was thinner in the brains of autistic people than in controls, consistent with decreased functional anisotropy (FA) in some frontal areas (Sundaram et al., 2008). The above findings demonstrate that the connectivity status in autism varies depending on cortical region.

The changes in axons below the ACC are of special interest for several reasons. To begin with, in non-human primates the ACC has the most widespread connections with neighboring prefrontal cortices (Barbas et al., 1999). The ACC may exercise its critical role in allocating attention through its normally extensive influence on the rest of the prefrontal cortex. Further, in non-human primates, excitatory pathways from the ACC innervate not only excitatory neurons in LPFC, but also a smaller but significant proportion of inhibitory neurons. Importantly, pathways from ACC form large and efficient synapses with inhibitory neurons in LPFC, and innervate preferentially the neurochemical class of inhibitory neurons labeled for calbindin (Medalla and Barbas, 2009, 2010), which are suited to reduce neural noise and enhance signal (Constantinidis et al., 2002; Wang et al., 2004). The exuberance of axons that connect the ACC with LPFC over short or medium distances may help explain why people with autism focus on a stimulus and have difficulty in orienting to other stimuli in the environment when needed. The problems in shifting attention are universal among people with autism, who are otherwise heterogeneous with regard to language acquisition, or the presence or absence of mental retardation or epilepsy (Zikopoulos and Barbas, 2010).

On the other hand, there is general agreement that pathways that travel over long distances are weak in autism, based on a variety of physiological and structural data (Courchesne and Pierce, 2005; Lepagnol-Bestel et al., 2008; Zikopoulos and Barbas, 2010; Muller et al., 2011; Schipul et al., 2011; Just et al., 2012), including findings at the level of single axons (Zikopoulos and Barbas, 2010). This consistent finding in autism likely contributes to the incongruence of cortical rhythms that engage distant cortices in autism (Thatcher et al., 2009; Lai et al., 2010; Khan et al., 2013). The physiological changes within large scale networks may help explain why people with autism have difficulty in shifting attention from one stimulus to another as the situation demands. In non-human primates, long-distance pathways are sparse in comparison with short-range pathways, which account for about 80% of connections (Barbas, 1988; Hilgetag et al., 2000; Hilgetag and Grant, 2000; Hilgetag and Kaiser, 2004; Barbas et al., 2005a). Nevertheless, long-distance pathways have considerable influence on the cortex. The prefrontal cortex, in particular, relies on sparse long-distance pathways for sensory input. Long-distance pathways also include interhemispheric connections, which have a critical role for synthesizing information across the commissures for a large variety of cognitive tasks, including language. In non-human primates, connections across the two hemispheres are less dense than connections within one hemisphere but involve just as many areas as the ipsilateral, at least for the prefrontal cortex (Barbas et al., 2005a). Contralateral pathways are also severely compromised in autism (Alexander et al., 2007; Just et al., 2007; Frazier and Hardan, 2009; Jou et al., 2010; Anderson et al., 2011b; Cantlon et al., 2011; Casanova et al., 2011; Fame et al., 2011; Schipul et al., 2011). In view of their functional significance and lower density, even small changes in long-range connections in autism likely have devastating effects on function.

In conclusion, areas are affected in varied ways in their connections in autism (Figure 5). In the superficial white matter below ACC, there is exuberance of short- or medium-range axons that link areas over short or medium distances. The white matter below lateral areas 9 and 46 shows no differences in axon density. On the other hand, in the superficial white matter below orbitofrontal area 11 the myelin is thinner, suggesting weak local connectivity. In the deep white matter below ACC there is a paucity of large axons that connect it with distant sensory and association areas. Pathology in ACC, which has a key role in attention, suggests that it may be the epicenter for abnormalities elsewhere, resulting in deficits in attention—excessive focusing on one stimulus or thought, and inability to disengage and attend to other stimuli flexibly. The deficits in ACC are consistent with the universal problems in attention in people with autism regardless of the severity of symptoms.

### A testable biological model relates structural axon features in autism to development

Why are thin and medium axons in excess just below the ACC, large axons in short supply in long-distance pathways, and myelin is insufficient in orbital area 11? Are these disparate findings independent or related? Autism is a disorder with its roots in development and to begin to sort out what may go awry with connections it is necessary to consider the development of affected areas (Figure 5). Let us first consider the ACC, which appears to have more than its share of deficiencies in autism. In non-human primates the ACC develops early in ontogeny (Rakic, 2002). When migrating neurons take their position in the cortex they extend axons that branch to connect with other areas. Several proteins expressed in development are critical for axon growth and guidance. One of these proteins is GAP-43, which is expressed at high levels in all areas during development (Milosevic et al., 1995; Kanazir et al., 1996; Oishi et al., 1998). In adult brains GAP-43 is expressed in significant levels only in some areas, albeit less than in development, and the ACC is one such region. The continued presence of GAP-43 into normal adulthood may help explain the numerous pathways that connect the ACC with neighboring areas, as seen in normal non-human primates (Barbas et al., 1999).

In contrast to the early migration of neurons in ACC, myelination begins much later, and is nearly as late as the last myelinating lateral prefrontal areas (Flechsig, 1901; Von Bonin, 1950; Yakovlev and Lecours, 1967; Hasegawa et al., 1992). Why are two developmental processes so much separated in time in the ACC? It turns out that GAP-43 and myelin proteins inhibit each other and consequently there is an inverse relationship between GAP-43 expression and myelination (Kapfhammer and Schwab, 1994; Benowitz and Routtenberg, 1997). Axons first elongate and then myelinate. The onset and duration of myelination varies among cortical areas, starting prenatally, gradually increasing postnatally, and continuing throughout childhood in most prefrontal cortices (Flechsig, 1901; Von Bonin, 1950; Yakovlev and Lecours, 1967; Benes, 1989; Paus et al., 1999, 2001; Levitt, 2003; Suzuki et al., 2003). The differences in development and myelination among areas may help explain why areas are not equally affected in autism.

In the brains of adults with autism just below ACC, GAP-43 is expressed in more than double the number of axons than in normal controls (Zikopoulos and Barbas, 2010). If expression of GAP-43 is also higher in children with autism that would help explain the exuberant branching of axons below ACC in adults. We used data from development and our findings from *post-mortem* brains from adults with autism to construct a biological model (Zikopoulos and Barbas, 2010). The model shows in broad terms the likely fate of axons and their branching and myelination based strictly on the sequence of developmental events in non-human primates and humans. A high level of GAP-43 in ACC, which develops early (Rakic, 2002), promotes axon growth and branching. The selective increase in medium and thin axons in the superficial white matter below the ACC is explained by the exposure of axons to GAP-43, which is highest at the growing end of axons, mediating branching as axons enter or leave the white matter to link nearby areas. This pattern is expected to increase the density of medium and thin axons. The model shows that myelination should not be affected, because the ACC myelinates very late (Flechsig, 1901; Von Bonin, 1950; Yakovlev and Lecours, 1967), when GAP-43 level drops relative to its expression early in development.

Development takes a different temporal course in OFC, where there is no excessive branching of axons but the myelin is thinner in the brains of autistic adults (Zikopoulos and Barbas, 2010). In OFC, neurons normally migrate to the cortex later than in ACC, but myelinate earlier, effectively shortening the interval between neuronal migration and axonal myelination. Based on these developmental events, the model predicts that a small increase in GAP-43 in OFC in development can affect myelination but not axon branching, as seen in the brains of adults with autism (Zikopoulos and Barbas, 2010). In lateral prefrontal areas 9 and 46, neurogenesis and migration are completed much later (Rakic, 2002), when levels of GAP-43 are comparatively low, which helps explain why neither axon branching nor myelination are affected in adults with autism (Zikopoulos and Barbas, 2010).

The predictions of our biological model, which is testable, are bolstered by recent genetic studies that have associated single nucleotide polymorphisms in the GAP-43 gene with autism (Allen-Brady et al., 2009), and identified its extended chromosomal region as an autism risk locus (Trikalinos et al., 2006; Szatmari et al., 2007). In addition, studies in mice have shown that wide changes in the levels of GAP-43 can lead to autistic-like behaviors, including learning disability and stereotypical behaviors (Routtenberg et al., 2000; Zaccaria et al., 2010).

Atypical GAP-43 levels in autism may, therefore, help explain the exuberance of short-range pathways below ACC, which leads to intrinsic overconnectivity in the frontal lobe (Courchesne and Pierce, 2005). Importantly, based on the late onset and completion of the development of connections between distant cortices, high levels of GAP-43 in ACC may also help explain the weakened long-distance connections that course in the deep white matter below ACC. Reduction in strength of long-distance pathways that course through the deep white matter in autism may be secondary to the excessive short-range connections, which develop first, reach their targets fast, and occupy sites that normally would be available to the sparser long-distance pathways (Zikopoulos and Barbas, 2010). Pathways that reach the ACC from a long distance thus may be at a competitive disadvantage, not only because they develop late, but also because their axons must continue to elongate to reach and form synapses in the prefrontal cortex.

In conclusion, using the distinct findings in ACC, orbitofrontal and lateral prefrontal areas and their relationship to developmental events, including neuronal migration, axonal branching in the presence of GAP-43, and myelination, a biological model can help explain the varied effects within the frontal lobe. The findings suggest overconnectivity of the ACC with nearby areas, long-distance disconnection, weakening of nearby connections of the OFC, and sparing of axonal structure in lateral prefrontal areas 9 and 46. However, even though none of the changes seen in axons below ACC or orbitofrontal area 11 were evident below prefrontal areas 9 and 46, the interlinkage of these areas suggests that they do not remain unscathed. Indeed, the relationship of axon types was seriously altered among prefrontal areas, suggesting widespread repercussions beyond the immediate areas affected.

In line with the above findings, the increased density of spines of the late-developing neurons in the superficial layers of lateral prefrontal areas may help accommodate excessive feedback from ACC in autism. Moreover, lateral prefrontal areas appear to have reduced PV/CB ratio, due to fewer PV inhibitory neurons, which also develop later than CB neurons in animals and humans. Future studies with more cases are needed to investigate if the ratio of distinct inhibitory neurons is altered in autism and may help explain the changes seen in GABA receptors. Combined, these findings provide converging information about the developmental timeline of ASD, pointing to a critical perinatal period for the emergence of axon pathology and neural communication deficits in autism.

## Materials and methods

### Tissue preparation

*Post-mortem* prefrontal brain tissue was obtained from the Harvard Brain Tissue Resource Center through the Autism Tissue Program from two autistic male adults and two typically developed, age-matched, male controls, ages 30–44 years. The selection of cases used was based on tissue availability of cases with closely matched characteristics, including *post-mortem* interval (Table 1), and period of storage of tissue in formalin (mean ± standard deviation = 137 ± 37 months), which minimized variability of tissue immunolabeling and shrinkage. The study was approved by the Institutional Review Board of Boston University. The diagnosis of autism was based on the Autism Diagnostic Interview-Revised (ADI-R) in both cases (Table 1). Clinical characteristics are summarized in Table 1. We excised small blocks (~2 × 3 cm) of matched frontal coronal tissue slabs (~1 cm thick), containing gray and white matter from DLPFC area 9 (Figure 3) based on the human brain atlas from the Autism Tissue Portal (www.atpportal.org) and (Von Economo, 2009, re-issued), and additional cytoarchitectonic studies of human prefrontal cortex (Selemon et al., 1998; Stark et al., 2004; Miguel-Hidalgo et al., 2006). We matched all samples to minimize variability and maximize statistical power. To ensure adequate preservation of the tissue the blocks were stored at −20°C in anti-freeze solution (30% ethylene glycol, 30% glycerol, 0.05% azide in PB). The blocks were rinsed in 0.1 M PB and cut coronally in series of adjacent sections (50 μm) on a vibratome (Pelco, series 1000).

### Immunohistochemistry

We used standard immunohistochemical procedures to label inhibitory neurons, as described (e.g., Barbas et al., 2005b; Zikopoulos and Barbas, 2006, 2007). Briefly, free-floating sections (50 μm thick) were treated with 1% H_2_O_2_ aqueous solution to suppress endogenous peroxidase activity, followed by 0.05 M glycine in 0.01 M phosphate buffered saline (PBS), pH: 7.4, to reduce cross-linking of lipids due to fixation. Tissue was placed in blocking solution of 0.3% Triton-X, 5% bovine serum albumin (BSA), 5% normal goat serum (NGS) in PBS, and then incubated in mouse monoclonal antibody (0.3% Triton-X in PBS) against CB, or PV, (1:2000, Swant). The sections were then incubated with a secondary biotinylated anti-mouse antibody (1:200 in PBS with 0.1% Triton-X; Vector), followed by avidin-biotin-peroxidase solution (Vector ABC Elite kit). We visualized positive neurons by the peroxidase-catalyzed polymerization of 0.05% 3,3-diaminobenzidine tetrahydrochloride (DAB; Zymed Laboratories) in 0.01% H_2_O_2_ buffer solution (pH, 7.5). After binding of the primary antibodies some sections were rinsed in PBS, incubated for 4 h with goat anti-mouse secondary antibodies conjugated with the fluorescent probes Alexa Fluor 488 (green) or 568 (red; 1:100; Invitrogen) and rinsed with PBS. To test for non-specific labeling we performed control experiments with sections adjacent to the experimental, omitting the primary antibodies, and incubating with secondary antisera. A small number of CB+ neurons in the cortex are pyramidal, but their labeling is minimized by using a monoclonal antibody (Gonzalez-Albo et al., 2001; and personal observations). In addition, we can morphologically identify these neurons, since they are larger and have spiny dendrites as opposed to smooth, small bipolar inhibitory CB neurons.

### Stereological analysis—light and confocal microscopy

We estimated the laminar density of labeled PV and CB inhibitory neurons as well as total neuronal density in tissue blocks of similar size and volume of DLPFC area 9 using the stereological method of the optical fractionator (Gundersen, 1986; Howard and Reed, 1998) and specific software (StereoInvestigator; Microbrightfield) under the microscope at high magnification (×400), as we have described (e.g., Dombrowski et al., 2001; Zikopoulos and Barbas, 2006, 2010). For microscopic analyses we used a minimum of three sections from one series of coronal sections (50 μm thick) from each case. To estimate the number of neurons we first measured the thickness of each section, and used StereoInvestigator to set a guard zone at the bottom and top of each section to correct for objects plucked during sectioning; the disector thickness was thus smaller than the thickness of the section (Gundersen, 1986; West et al., 1991; Howard and Reed, 1998). The sampling fraction was 1/50 of the total volume of the area examined. The use of uniform random sampling ensured that every part of the area examined had the same chance of being included in the sample. The estimated numbers of neurons and the volumes of the corresponding layers (estimated with the Cavalieri method) were divided to assess relative density of label. In all experiments we stained one series of sections for Nissl (thionin) to place cytoarchitectonic borders. The section surface, the cytoarchitectonic borders of areas of interest, and layers, were outlined with the aid of a commercial computerized microscope system and motorized stage at a magnification ×400.

It should be noted here that the densities we report are relative, not absolute, since we did not apply a correction factor to account for inevitable tissue shrinkage during prolonged fixation and immunohistochemical processing. Variability due to tissue shrinkage was likely minimal because the period of storage of tissue in fixative was comparable across cases, and brain sections were simultaneously processed, using a standardized protocol, under identical conditions. This resulted in comparable shrinkage of tissue due to processing, mounting, and air drying, which was minimal in the X and Y dimensions (~2%) and within expected levels in the Z dimension (~65%), as reported previously (Dombrowski et al., 2001). Matching of normal and autistic brain sections and simultaneous processing under identical conditions, minimized experimental variability and made it possible to use a small number of brains, as each brain yields a large amount of data. This is particularly critical with the use of a rare and valuable resource of human brain tissue.

### Statistical analysis

We gathered data blind to condition and cortical region. Random codes for cases and images were broken after completion of each part of the study, as described (Zikopoulos and Barbas, 2010). In all cases data collection was performed by at least two investigators. Values obtained from the two independent measures were highly correlated (Pearson *R* = 0.97, *p* = 0.001). Data distributions for continuous variables were not significantly different from normal as determined by the Kolmogorov–Smirnov test, and thus allowed the use of parametric statistics. Data were evaluated with Statistica (StatSoft, Tulsa, OK), through a two-tailed *t*-test. For all analyses *p*-values <0.05 were taken as statistically significant.

## Author contributions

Basilis Zikopoulos and Helen Barbas designed the experiments. Basilis Zikopoulos performed and analyzed the experiments. Basilis Zikopoulos and Helen Barbas prepared the manuscript.

### Conflict of interest statement

The authors declare that the research was conducted in the absence of any commercial or financial relationships that could be construed as a potential conflict of interest.

## Appendix

### Abbreviations

ACC: Anterior cingulate cortex
ASD: Autism spectrum disorders
CB: Calbindin
CC: Corpus callosum
CR: Calretinin
cs: Central sulcus
DLPFC: Dorsolateral prefrontal cortex
DWM: Deep white matter
FFA: Fusiform face area
FFG: Fusiform gyrus
GAP-43: Growth axon protein 43 KDa
ifs: Inferior frontal sulcus
LPFC: Lateral prefrontal cortex
M1: Primary motor cortex
OFC: Orbitofrontal cortex
PCC: Posterior cingulate cortex
PFC: Prefrontal cortex
PV: Parvalbumin
sfs: Superior frontal sulcus
SMA: Supplementary motor area
SWM: Superficial white matter.

## References

[B1] Abbott L. F. Chance F. S. (2005). Drivers and modulators from push-pull and balanced synaptic input. Prog. Brain Res. 149, 147–155 10.1016/S0079-6123(05)49011-1 16226582

[B2] Abrams D. A. Lynch C. J. Cheng K. M. Phillips J. Supekar K. Ryali S. (2013). Underconnectivity between voice-selective cortex and reward circuitry in children with autism. Proc. Natl. Acad. Sci. U.S.A. 110, 12060–12065 10.1073/pnas.1302982110 23776244 PMC3718181

[B3] Ahmed B. Anderson J. C. Martin K. A. Nelson J. C. (1997). Map of the synapses onto layer 4 basket cells of the primary visual cortex of the cat. J. Comp. Neurol. 380, 230–242 10.1002/(SICI)1096-9861(19970407)380:2<230::AID-CNE6>3.0.Co;2-4 9100134

[B4] Alcantara S. Ferrer I. Soriano E. (1993). Postnatal development of parvalbumin and calbindin D28K immunoreactivities in the cerebral cortex of the rat. Anat. Embryol. 188, 63–73 10.1007/BF00191452 8214625

[B5] Alexander A. L. Lee J. E. Lazar M. Boudos R. Dubray M. B. Oakes T. R. (2007). Diffusion tensor imaging of the corpus callosum in Autism. Neuroimage 34, 61–73 10.1016/j.neuroimage.2006.08.032 17023185

[B6] Allen-Brady K. Miller J. Matsunami N. Stevens J. Block H. Farley M. (2009). A high-density SNP genome-wide linkage scan in a large autism extended pedigree. Mol. Psychiatry 14, 590–600 10.1038/mp.2008.14 18283277

[B7] Alonso-Nanclares L. White E. L. Elston G. N. Defelipe J. (2004). Synaptology of the proximal segment of pyramidal cell basal dendrites. Eur. J. Neurosci. 19, 771–776 10.1111/j.0953-816X.2003.03166.x 14984428

[B8] Amaral D. G. Schumann C. M. Nordahl C. W. (2008). Neuroanatomy of autism. Trends Neurosci. 31, 137–145 10.1016/j.tins.2007.12.005 18258309

[B9] Anagnostou E. Taylor M. (2011). Review of neuroimaging in autism spectrum disorders: what we have learned and where we go from here. Mol. Autism 2:4 10.1186/2040-2392-2-4 21501488 PMC3102613

[B10] Anderson J. C. Kennedy H. Martin K. A. C. (2011a). Pathways of attention: synaptic relationships of frontal eye field to V4, lateral intraparietal cortex, and area 46 in macaque monkey. J. Neurosci. 31, 10872–10881 10.1523/JNEUROSCI.0622-11.2011 21795539 PMC6623081

[B11] Anderson J. S. Druzgal T. J. Froehlich A. Dubray M. B. Lange N. Alexander A. L. (2011b). Decreased interhemispheric functional connectivity in autism. Cereb. Cortex 21, 1134–1146 10.1093/Cercor/bhq190 20943668 PMC3077433

[B12] Anderson J. C. Martin K. A. (2009). The synaptic connections between cortical areas V1 and V2 in macaque monkey. J. Neurosci. 29, 11283–11293 10.1523/JNEUROSCI.5757-08.2009 19741135 PMC6665918

[B13] Assaf M. Jagannathan K. Calhoun V. D. Miller L. Stevens M. C. Sahl R. (2010). Abnormal functional connectivity of default mode sub-networks in autism spectrum disorder patients. Neuroimage 53, 247–256 10.1016/j.neuroimage.2010.05.067 20621638 PMC3058935

[B14] Atladottir H. O. Pedersen M. G. Thorsen P. Mortensen P. B. Deleuran B. Eaton W. W. (2009). Association of family history of autoimmune diseases and autism spectrum disorders. Pediatrics 124, 687–694 10.1542/peds.2008-2445 19581261

[B15] Avino T. A. Hutsler J. J. (2010). Abnormal cell patterning at the cortical gray-white matter boundary in autism spectrum disorders. Brain Res. 1360, 138–146 10.1016/j.brainres.2010.08.091 20816758

[B16] Avino T. A. Wojcik C. Mann A. Hutsler J. J. (2012). “Morphological analysis of dendritic spines on cortical pyramidal cells in ASD,” in 2012 International Meeting for Autism Research (Toronto, ON).

[B17] Bachevalier J. Loveland K. A. (2006). The orbitofrontal-amygdala circuit and self-regulation of social-emotional behavior in autism. Neurosci. Biobehav. Rev. 30, 97–117 10.1016/j.neubiorev.2005.07.002 16157377

[B18] Badre D. Wagner A. D. (2004). Selection, integration, and conflict monitoring; assessing the nature and generality of prefrontal cognitive control mechanisms. Neuron 41, 473–487 10.1016/S0896-6273(03)00851-1 14766185

[B19] Bailey A. Luthert P. Dean A. Harding B. Janota I. Montgomery M. (1998). A clinicopathological study of autism. Brain 121(Pt 5), 889–905 10.1093/brain/121.5.889 9619192

[B20] Barbas H. (1986). Pattern in the laminar origin of corticocortical connections. J. Comp. Neurol. 252, 415–422 10.1002/cne.902520310 3793985

[B21] Barbas H. (1988). Cortical projections to orbitofrontal limbic cortices in the rhesus monkey. Neurosci. Abstr. 14, 922

[B22] Barbas H. (1993). Organization of cortical afferent input to orbitofrontal areas in the rhesus monkey. Neuroscience 56, 841–864 10.1016/0306-4522(93)90132-Y 8284038

[B23] Barbas H. (2000a). Complementary role of prefrontal cortical regions in cognition, memory and emotion in primates. Adv. Neurol. 84, 87–110 11091860

[B24] Barbas H. (2000b). Connections underlying the synthesis of cognition, memory, and emotion in primate prefrontal cortices. Brain Res. Bull. 52, 319–330 10.1016/S0361-9230(99)00245-2 10922509

[B25] Barbas H. Mesulam M. M. (1985). Cortical afferent input to the principalis region of the rhesus monkey. Neuroscience 15, 619–637 10.1016/0306-4522(85)90064-8 4069349

[B26] Barbas H. Rempel-Clower N. (1997). Cortical structure predicts the pattern of corticocortical connections. Cereb. Cortex 7, 635–646 10.1093/cercor/7.7.635 9373019

[B27] Barbas H. Zikopoulos B. (2006). “Sequential and parallel circuits for emotional processing in primate orbitofrontal cortex,” in The Orbitofrontal Cortex, eds David Z. Scott R. (Oxford: Oxford University Press), 57–91

[B28] Barbas H. Zikopoulos B. (2007). The prefrontal cortex and flexible behavior. Neuroscientist 13, 532–545 10.1177/1073858407301369 17901261 PMC2855184

[B29] Barbas H. Ghashghaei H. Dombrowski S. M. Rempel-Clower N. L. (1999). Medial prefrontal cortices are unified by common connections with superior temporal cortices and distinguished by input from memory-related areas in the rhesus monkey. J. Comp. Neurol. 410, 343–367 10.1002/(SICI)1096-9861(19990802)410:3<343::AID-CNEI>3.0.CO;2-1 10404405

[B30] Barbas H. Hilgetag C. C. Saha S. Dermon C. R. Suski J. L. (2005a). Parallel organization of contralateral and ipsilateral prefrontal cortical projections in the rhesus monkey. BMC Neurosci. 6:32 10.1186/1471-2202-6-32 15869709 PMC1134662

[B31] Barbas H. Medalla M. Alade O. Suski J. Zikopoulos B. Lera P. (2005b). Relationship of prefrontal connections to inhibitory systems in superior temporal areas in the rhesus monkey. Cereb. Cortex 15, 1356–1370 10.1093/cercor/bhi018 15635060

[B32] Barbas H. Zikopoulos B. Timbie C. (2011). Sensory pathways and emotional context for action in primate prefrontal cortex. Biol. Psychiatry 69, 1133–1139 10.1016/j.biopsych.2010.08.008 20889144

[B33] Barnea-Goraly N. Kwon H. Menon V. Eliez S. Lotspeich L. Reiss A. L. (2004). White matter structure in autism: preliminary evidence from diffusion tensor imaging. Biol. Psychiatry 55, 323–326 10.1016/j.biopsych.2003.10.022 14744477

[B34] Baron-Cohen S. (1991). The development of a theory of mind in autism: deviance and delay? Psychiatr. Clin. North Am. 14, 33–51 2047331

[B35] Bauman M. L. Kemper T. L. (2005). Neuroanatomic observations of the brain in autism: a review and future directions. Int. J. Dev. Neurosci. 23, 183–187 10.1016/j.ijdevneu.2004.09.006 15749244

[B36] Bauman M. Kemper T. L. (1985). Histoanatomic observations of the brain in early infantile autism. Neurology 35, 866–874 10.1212/WNL.35.6.866 4000488

[B37] Becker K. G. Schultz S. T. (2010). Similarities in features of autism and asthma and a possible link to acetaminophen use. Med. Hypotheses 74, 7–11 10.1016/j.mehy.2009.08.033 19748189 PMC3261751

[B38] Belmonte M. K. Allen G. Beckel-Mitchener A. Boulanger L. M. Carper R. A. Webb S. J. (2004a). Autism and abnormal development of brain connectivity. J. Neurosci. 24, 9228–9231 10.1523/JNEUROSCI.3340-04.2004 15496656 PMC6730085

[B39] Belmonte M. K. Cook E. H. Anderson G. M. Rubenstein J. L. R. Greenough W. T. Beckel-Mitchener A. (2004b). Autism as a disorder of neural information processing: directions for research and targets for therapy. Mol. Psychiatry 9, 646–663 15037868 10.1038/sj.mp.4001499

[B40] Benes F. M. (1989). Myelination of cortical-hippocampal relays during late adolescence. Schizophr. Bull. 15, 585–593 10.1093/schbul/15.4.585 2623440

[B41] Benes F. M. Vincent S. L. Todtenkopf M. (2001). The density of pyramidal and nonpyramidal neurons in anterior cingulate cortex of schizophrenic and bipolar subjects. Biol. Psychiatry 50, 395–406 10.1016/S0006-3223(01)01084-8 11566156

[B42] Benowitz L. I. Routtenberg A. (1997). GAP-43: an intrinsic determinant of neuronal development and plasticity. Trends Neurosci. 20, 84–91 10.1016/S0166-2236(96)10072-2 9023877

[B43] Berger B. Trottier S. Verney C. Gaspar P. Alvarez C. (1988). Regional and laminar distribution of the dopamine and serotonin innervation in the macaque cerebral cortex: a radioautographic study. J. Comp. Neurol. 273, 99–119 10.1002/cne.902730109 3209731

[B44] Bernardi S. Anagnostou E. Shen J. Kolevzon A. Buxbaum J. D. Hollander E. (2011). *In vivo* (1)H-magnetic resonance spectroscopy study of the attentional networks in autism. Brain Res. 1380, 198–205 10.1016/j.brainres.2010.12.057 21185269 PMC3073642

[B45] Bigler E. D. Mortensen S. Neeley E. S. Ozonoff S. Krasny L. Johnson M. (2007). Superior temporal gyrus, language function, and autism. Dev. Neuropsychol. 31, 217–238 10.1080/87565640701190841 17488217

[B46] Blatt G. J. Fatemi S. H. (2011). Alterations in GABAergic biomarkers in the autism brain. Anat. Rec. 294, 1646–1652 10.1002/ar.21252 21901839 PMC3190183

[B47] Blatt G. J. Fitzgerald C. M. Guptill J. T. Booker A. B. Kemper T. L. Bauman M. L. (2001). Density and distribution of hippocampal neurotransmitter receptors in autism: an autoradiographic study. J. Autism Dev. Disord. 31, 537–543 10.1023/A:1013238809666 11814263

[B48] Blaxill M. F. (2004). What's going on? The question of time trends in autism. Public Health Rep. 119, 536–551 10.1016/j.phr.2004.09.003 15504445 PMC1497666

[B49] Borgers C. Kopell N. (2003). Synchronization in networks of excitatory and inhibitory neurons with sparse, random connectivity. Neural Comput. 15, 509–538 10.1162/089976603321192059 12620157

[B50] Borgers C. Epstein S. Kopell N. J. (2008). Gamma oscillations mediate stimulus competition and attentional selection in a cortical network model. Proc. Natl. Acad. Sci. U.S.A. 105, 18023–18028 10.1073/pnas.0809511105 19004759 PMC2584712

[B51] Brown J. S. Jr. (2009). Effects of bisphenol-A and other endocrine disruptors compared with abnormalities of schizophrenia: an endocrine-disruption theory of schizophrenia. Schizophr. Bull. 35, 256–278 10.1093/schbul/sbm147 18245062 PMC2643957

[B52] Bunce J. G. Barbas H. (2011). Prefrontal pathways target excitatory and inhibitory systems in memory-related medial temporal cortices. Neuroimage 55, 1461–1474 10.1016/j.neuroimage.2011.01.064 21281716 PMC3500621

[B53] Buxhoeveden D. P. Semendeferi K. Buckwalter J. Schenker N. Switzer R. Courchesne E. (2006). Reduced minicolumns in the frontal cortex of patients with autism. Neuropathol. Appl. Neurobiol. 32, 483–491 10.1111/j.1365-2990.2006.00745.x 16972882

[B54] Buzsaki G. Draguhn A. (2004). Neuronal oscillations in cortical networks. Science 304, 1926–1929 10.1126/science.1099745 15218136

[B55] Cantlon J. F. Davis S. W. Libertus M. E. Kahane J. Brannon E. M. Pelphrey K. A. (2011). Inter-parietal white matter development predicts numerical performance in young children. Learn. Individ. Differ. 21, 672–680 10.1016/j.lindif.2011.09.003 22180720 PMC3240671

[B56] Cao F. Yin A. Wen G. Sheikh A. M. Tauqeer Z. Malik M. (2012). Alteration of astrocytes and Wnt/beta-catenin signaling in the frontal cortex of autistic subjects. J. Neuroinflammation 9, 223 10.1186/1742-2094-9-223 22999633 PMC3544729

[B57] Carper R. A. Courchesne E. (2005). Localized enlargement of the frontal cortex in early autism. Biol. Psychiatry 57, 126–133 10.1016/j.biopsych.2004.11.005 15652870

[B58] Carper R. A. Moses P. Tigue Z. D. Courchesne E. (2002). Cerebral lobes in autism: early hyperplasia and abnormal age effects. Neuroimage 16, 1038–1051 10.1006/nimg.2002.1099 12202091

[B59] Casanova M. F. (2004). White matter volume increase and minicolumns in autism. Ann. Neurol. 56, 453 10.1002/ana.20196 15349878

[B60] Casanova M. F. (2007). The neuropathology of autism. Brain Pathol. 17, 422–433 10.1111/j.1750-3639.2007.00100.x 17919128 PMC8095561

[B61] Casanova M. F. Buxhoeveden D. P. Brown C. (2002a). Clinical and macroscopic correlates of minicolumnar pathology in autism. J. Child Neurol. 17, 692–695 10.1177/088307380201700908 12503647

[B62] Casanova M. F. Buxhoeveden D. P. Switala A. E. Roy E. (2002b). Minicolumnar pathology in autism. Neurology 58, 428–432 10.1212/WNL.58.3.428 11839843

[B63] Casanova M. F. Buxhoeveden D. Gomez J. (2003). Disruption in the inhibitory architecture of the cell minicolumn: implications for autism. Neuroscientist 9, 496–507 10.1177/1073858403253552 14678582

[B64] Casanova M. F. El Baz A. Elnakib A. Switala A. E. Williams E. L. Williams D. L. (2011). Quantitative analysis of the shape of the corpus callosum in patients with autism and comparison individuals. Autism 15, 223–238 10.1177/1362361310386506 21363871 PMC3349188

[B65] Casanova M. F. van Kooten I. A. Switala A. E. van Engeland H. Heinsen H. Steinbusch H. W. (2006). Minicolumnar abnormalities in autism. Acta Neuropathol. 112, 287–303 10.1007/s00401-006-0085-5 16819561

[B66] Chao H. T. Chen H. Samaco R. C. Xue M. Chahrour M. Yoo J. (2010). Dysfunction in GABA signalling mediates autism-like stereotypies and Rett syndrome phenotypes. Nature 468, 263–269 10.1038/nature09582 21068835 PMC3057962

[B67] Cheon K. A. Kim Y. S. Oh S. H. Park S. Y. Yoon H. W. Herrington J. (2011). Involvement of the anterior thalamic radiation in boys with high functioning autism spectrum disorders: a Diffusion Tensor Imaging study. Brain Res. 1417, 77–86 10.1016/j.brainres.2011.08.020 21890117

[B68] Chow C. C. White J. A. Ritt J. Kopell N. (1998). Frequency control in synchronized networks of inhibitory neurons. J. Comput. Neurosci. 5, 407–420 10.1023/A:1008889328787 9877022

[B69] Collins A. L. Ma D. Whitehead P. L. Martin E. R. Wright H. H. Abramson R. K. (2006). Investigation of autism and GABA receptor subunit genes in multiple ethnic groups. Neurogenetics 7, 167–174 10.1007/s10048-006-0045-1 16770606 PMC1513515

[B70] Conde F. Lund J. S. Jacobowitz D. M. Baimbridge K. G. Lewis D. A. (1994). Local circuit neurons immunoreactive for calretinin, calbindin D- 28k or parvalbumin in monkey prefrontal cortex: distribution and morphology. J. Comp. Neurol. 341, 95–116 10.1002/cne.903410109 8006226

[B71] Constantinidis C. Williams G. V. Goldman-Rakic P. S. (2002). A role for inhibition in shaping the temporal flow of information in prefrontal cortex. Nat. Neurosci. 5, 175–180 10.1038/nn799 11802172

[B72] Courchesne E. Pierce K. (2005). Why the frontal cortex in autism might be talking only to itself: local over-connectivity but long-distance disconnection. Curr. Opin. Neurobiol. 15, 225–230 10.1016/j.conb.2005.03.001 15831407

[B73] Courchesne E. Campbell K. Solso S. (2011a). Brain growth across the life span in autism: age-specific changes in anatomical pathology. Brain Res. 1380, 138–145 20920490 10.1016/j.brainres.2010.09.101 PMC4500507

[B74] Courchesne E. Mouton P. R. Calhoun M. E. Semendeferi K. Ahrens-Barbeau C. Hallet M. J. (2011b). Neuron number and size in prefrontal cortex of children with autism. JAMA 306, 2001–2010 10.1001/jama.2011.1638 22068992

[B75] Courchesne E. Pierce K. Schumann C. M. Redcay E. Buckwalter J. A. Kennedy D. P. (2007). Mapping early brain development in autism. Neuron 56, 399–413 10.1016/j.neuron.2007.10.016 17964254

[B76] Croen L. A. Grether J. K. Yoshida C. K. Odouli R. Hendrick V. (2011). Antidepressant use during pregnancy and childhood autism spectrum disorders. Arch. Gen. Psychiatry. 68, 1104–1112 10.1001/archgenpsychiatry.2011.73 21727247

[B77] Croen L. A. Grether J. K. Yoshida C. K. Odouli R. Van de W. J. (2005). Maternal autoimmune diseases, asthma and allergies, and childhood autism spectrum disorders: a case-control study. Arch. Pediatr. Adolesc. Med. 159, 151–157 10.1001/archpedi.159.2.151 15699309

[B78] de Cock M. Maas Y. G. van de Bor M. (2012). Does perinatal exposure to endocrine disruptors induce autism spectrum and attention deficit hyperactivity disorders? Review. Acta Paediatr. 101, 811–818 10.1111/j.1651-2227.2012.02693.x 22458970

[B79] Defelipe J. (1997). Types of neurons, synaptic connections and chemical characteristics of cells immunoreactive for calbindin-D28K, parvalbumin and calretinin in the neocortex. J. Chem. Neuroanat. 14, 1–19 10.1016/S0891-0618(97)10013-8 9498163

[B80] Defelipe J. (1999). Chandelier cells and epilepsy. Brain 122(Pt 10), 1807–1822 10.1093/brain/122.10.1807 10506085

[B81] Defelipe J. Gonzalez-Albo M. C. Del Rio M. R. Elston G. N. (1999). Distribution and patterns of connectivity of interneurons containing calbindin, calretinin, and parvalbumin in visual areas of the occipital and temporal lobes of the macaque monkey. J. Comp. Neurol. 412, 515–526 10.1002/(SICI)1096-9861(19990927)412:3<515::AID-CNE10>3.0.CO;2-1 10441237

[B82] Defelipe J. Hendry S. H. Jones E. G. (1989a). Synapses of double bouquet cells in monkey cerebral cortex visualized by calbindin immunoreactivity. Brain Res. 503, 49–54 10.1016/0006-8993(89)91702-2 2611658

[B83] Defelipe J. Hendry S. H. Jones E. G. (1989b). Visualization of chandelier cell axons by parvalbumin immunoreactivity in monkey cerebral cortex. Proc. Natl. Acad. Sci. U.S.A. 86, 2093–2097 10.1073/pnas.86.6.2093 2648389 PMC286854

[B84] Defelipe J. Hendry S. H. Hashikawa T. Molinari M. Jones E. G. (1990). A microcolumnar structure of monkey cerebral cortex revealed by immunocytochemical studies of double bouquet cell axons. Neuroscience 37, 655–673 10.1016/0306-4522(90)90097-N 1701039

[B85] Denny J. B. (2006). Molecular mechanisms, biological actions, and neuropharmacology of the growth-associated protein GAP-43. Curr. Neuropharmacol. 4, 293–304 10.2174/157015906778520782 18654638 PMC2475799

[B86] Di Martino A. Kelly C. Grzadzinski R. Zuo X. N. Mennes M. Mairena M. A. (2011). Aberrant striatal functional connectivity in children with autism. Biol. Psychiatry 69, 847–856 10.1016/j.biopsych.2010.10.029 21195388 PMC3091619

[B87] Dichter G. S. Felder J. N. Bodfish J. W. (2009). Autism is characterized by dorsal anterior cingulate hyperactivation during social target detection. Soc. Cogn. Affect. Neurosci. 4, 215–226 10.1093/scan/nsp017 19574440 PMC2728636

[B88] Dombrowski S. M. Hilgetag C. C. Barbas H. (2001). Quantitative architecture distinguishes prefrontal cortical systems in the rhesus monkey. Cereb. Cortex 11, 975–988 10.1093/cercor/11.10.975 11549620

[B89] Douglas R. J. Martin K. A. (2004). Neuronal circuits of the neocortex. Annu. Rev. Neurosci. 27, 419–451 10.1146/annurev.neuro.27.070203.144152 15217339

[B90] Ehninger D. Silva A. J. (2011). Rapamycin for treating Tuberous sclerosis and Autism spectrum disorders. Trends Mol. Med. 17, 78–87 10.1016/j.molmed.2010.10.002 21115397 PMC3075964

[B91] Fame R. M. MacDonald J. L. Macklis J. D. (2011). Development specification, and diversity of callosal projection neurons. Trends Neurosci. 34, 41–50 10.1016/j.tins.2010.10.002 21129791 PMC3053014

[B92] Fatemi S. H. Folsom T. D. Reutiman T. J. Thuras P. D. (2009a). Expression of GABA(B) receptors is altered in brains of subjects with autism. Cerebellum 8, 64–69 10.1007/s12311-008-0075-3 19002745 PMC2732344

[B93] Fatemi S. H. Reutiman T. J. Folsom T. D. Thuras P. D. (2009b). GABA(A) receptor downregulation in brains of subjects with autism. J. Autism Dev. Disord. 39, 223–230 10.1007/s10803-008-0646-7 18821008 PMC2697059

[B94] Flechsig P. (1901). Developmental (myelogenetic) localisation of the cerebral cortex in the human subject. Lancet 158, 1027–1029 10.1016/S0140-6736(01)01429-5

[B95] Frazier T. W. Hardan A. Y. (2009). A meta-analysis of the corpus callosum in autism. Biol. Psychiatry 66, 935–941 10.1016/j.biopsych.2009.07.022 19748080 PMC2783565

[B96] Fullerton B. C. Pandya D. N. (2007). Architectonic analysis of the auditory-related areas of the superior temporal region in human brain. J. Comp. Neurol. 504, 470–498 10.1002/cne.21432 17701981

[B97] Gabbott P. L. A. Jays P. R. L. Bacon S. J. (1997). Calretinin neurons in human medial prefrontal cortex (areas 24a,b,c, 32', and 25). J. Comp. Neurol. 381, 389–410 10.1002/(SICI)1096-9861(19970519)381:4<389::AID-CNE1>30.CO;2-Z 9136798

[B98] Gabbott P. L. Bacon S. J. (1996). Local circuit neurons in the medial prefrontal cortex (areas 24a,b,c, 25 and 32) in the monkey: II. Quantitative areal and laminar distributions. J. Comp. Neurol. 364, 609–636 10.1002/(SICI)1096-9861(19960122)364:4<609::AID-CNE2>3.0.CO;2-7 8821450

[B99] Gandal M. J. Sisti J. Klook K. Ortinski P. I. Leitman V. Liang Y. (2012). GABAB-mediated rescue of altered excitatory-inhibitory balance, gamma synchrony and behavioral deficits following constitutive NMDAR-hypofunction. Transl. Psychiatry 2:e142 10.1038/tp.2012.69 22806213 PMC3410621

[B100] Garbern J. Y. Neumann M. Trojanowski J. Q. Lee V. M. Feldman G. Norris J. W. (2010). A mutation affecting the sodium/proton exchanger, SLC9A6, causes mental retardation with tau deposition. Brain 133, 1391–1402 10.1093/brain/awq071 20395263 PMC2859154

[B101] Garbett K. A. Hsiao E. Y. Kalman S. Patterson P. H. Mirnics K. (2012). Effects of maternal immune activation on gene expression patterns in the fetal brain. Transl. Psychiatry 2:e98 10.1038/tp.2012.24 22832908 PMC3337077

[B102] Gaspar P. Berger B. Febvret A. Vigny A. Henry J. P. (1989). Catecholamine innervation of the human cerebral cortex as revealed by comparative immunohistochemistry of tyrosine hydroxylase and dopamine-beta-hydroxylase. J. Comp. Neurol. 279, 249–271 10.1002/cne.902790208 2563268

[B103] Gehring W. J. Knight R. T. (2000). Prefrontal-cingulate interactions in action monitoring. Nat. Neurosci. 3, 516–520 10.1038/74899 10769394

[B104] Germuska M. Saha S. Fiala J. C. Barbas H. (2006). Synaptic distinction of laminar specific prefrontal-temporal pathways in primates. Cereb. Cortex 16, 865–875 10.1093/cercor/bhj030 16151179

[B105] Geschwind D. H. (2011). Genetics of autism spectrum disorders. Trends Cogn. Sci. 15, 409–416 10.1016/j.tics.2011.07.003 21855394 PMC3691066

[B106] Ghashghaei H. T. Barbas H. (2002). Pathways for emotions: interactions of prefrontal and anterior temporal pathways in the amygdala of the rhesus monkey. Neuroscience 115, 1261–1279 10.1016/S0306-4522(02)00446-3 12453496

[B107] Ghashghaei H. T. Hilgetag C. C. Barbas H. (2007). Sequence of information processing for emotions based on the anatomic dialogue between prefrontal cortex and amygdala. Neuroimage 34, 905–923 10.1016/j.neuroimage.2006.09.046 17126037 PMC2045074

[B108] Gilman S. R. Iossifov I. Levy D. Ronemus M. Wigler M. Vitkup D. (2011). Rare de novo variants associated with autism implicate a large functional network of genes involved in formation and function of synapses. Neuron 70, 898–907 10.1016/j.neuron.2011.05.021 21658583 PMC3607702

[B109] Girgis R. R. Minshew N. J. Melhem N. M. Nutche J. J. Keshavan M. S. Hardan A. Y. (2007). Volumetric alterations of the orbitofrontal cortex in autism. Prog. NeuroPsychopharmacol. Biol. Psychiatry 31, 41–45 10.1016/j.pnpbp.2006.06.007 16863674 PMC2888006

[B110] Gomot M. Bernard F. A. Davis M. H. Belmonte M. K. Ashwin C. Bullmore E. T. (2006). Change detection in children with autism: an auditory event-related fMRI study. Neuroimage 29, 475–484 10.1016/j.neuroimage.2005.07.027 16115783

[B111] Gonchar Y. Burkhalter A. (1999). Connectivity of GABAergic calretinin-immunoreactive neurons in rat primary visual cortex. Cereb. Cortex 9, 683–696 10.1093/cercor/9.7.683 10554991

[B112] Gonzalez-Albo M. C. Elston G. N. Defelipe J. (2001). The human temporal cortex: characterization of neurons expressing nitric oxide synthase, neuropeptides and calcium-binding proteins, and their glutamate receptor subunit profiles. Cereb. Cortex 11, 1170–1181 10.1093/cercor/11.12.1170 11709488

[B113] Granda B. Tabernero A. Tello V. Medina J. M. (2003). Oleic acid induces GAP-43 expression through a protein kinase C-mediated mechanism that is independent of NGF but synergistic with NT-3 and NT-4/5. Brain Res. 988, 1–8 10.1016/S0006-8993(03)03253-0 14519521

[B114] Grant S. Hilgetag C. C. (2005). Graded classes of cortical connections: quantitative analyses of laminar projections to motion areas of cat extrastriate cortex. Eur. J. Neurosci. 22, 681–696 10.1111/j.1460-9568.2005.04232.x 16101750 PMC1351360

[B115] Gundersen H. J. (1986). Stereology of arbitrary particles. A review of unbiased number and size estimators and the presentation of some new ones, in memory of William R. Thompson. J. Microsc. 143(Pt 1), 3–45 10.1111/j.1365-2818.1986.tb02764.x 3761363

[B116] Gupta A. Wang Y. Markram H. (2000). Organizing principles for a diversity of GABAergic interneurons and synapses in the neocortex. Science 287, 273–278 10.1126/science.287.5451.273 10634775

[B117] Guptill J. T. Booker A. B. Gibbs T. T. Kemper T. L. Bauman M. L. Blatt G. J. (2007). [3H]-flunitrazepam-labeled benzodiazepine binding sites in the hippocampal formation in autism: a multiple concentration autoradiographic study. J. Autism Dev. Disord. 37, 911–920 10.1007/s10803-006-0226-7 17019626

[B118] Hallmayer J. Cleveland S. Torres A. Phillips J. Cohen B. Torigoe T. (2011). Genetic heritability and shared environmental factors among twin pairs with autism. Arch. Gen. Psychiatry 68, 1095–1102 10.1001/archgenpsychiatry.2011.76 21727249 PMC4440679

[B119] Hardan A. Y. Girgis R. R. Lacerda A. L. T. Yorbik O. Kilpatiick M. Keshavan M. S. (2006). Magnetic resonance imaging study of the orbitofrontal cortex in autism. J. Child Neurol. 21, 866–871 10.1177/08830738060210100701 17005103

[B120] Hasegawa M. Houdou S. Mito T. Takashima S. Asanuma K. Ohno T. (1992). Development of myelination in the human fetal and infant cerebrum: a myelin basic protein immunohistochemical study. Brain Dev. 14, 1–6 10.1016/S0387-7604(12)80271-3 1375444

[B121] Haznedar M. M. Buchsbaum M. S. Licalzi E. M. Cartwright C. Hollander E. (2006). Volumetric analysis and three-dimensional glucose metabolic mapping of the striatum and thalamus in patients with autism spectrum disorders. Am. J. Psychiatry 163, 1252–1263 10.1176/appi.ajp.163.7.1252 16816232

[B122] Hendry S. H. C. Jones E. G. Emson P. C. Lawson D. E. M. Heizmann C. W. Streit P. (1989). Two classes of cortical GABA neurons defined by differential calcium binding protein immunoreactivities. Exp. Brain Res. 76, 467–472 10.1007/BF00247904 2767197

[B123] Herbert M. R. (2005). Large brains in autism: the challenge of pervasive abnormality. Neuroscientist 11, 417–440 10.1177/0091270005278866 16151044

[B124] Herbert M. R. Ziegler D. A. Deutsch C. K. O'Brien L. M. Lange N. Bakardjiev A. (2003). Dissociations of cerebral cortex, subcortical and cerebral white matter volumes in autistic boys. Brain 126, 1182–1192 10.1093/brain/awg110 12690057

[B125] Herbert M. R. Ziegler D. A. Makris N. Filipek P. A. Kemper T. L. Normandin J. J. (2004). Localization of white matter volume increase in autism and developmental language disorder. Ann. Neurol. 55, 530–540 10.1002/ana.20032 15048892

[B126] Hilgetag C. C. Barbas H. (2006). Role of mechanical factors in the morphology of the primate cerebral cortex. PLoS Comput. Biol. 2:e22 10.1371/journal.pcbi.0020022 16557292 PMC1409812

[B127] Hilgetag C. C. Grant S. (2000). Uniformity, specificity and variability of corticocortical connectivity. Philos. Trans. R. Soc. Lond. B Biol. Sci. 355, 7–20 10.1098/rstb.2000.0546 10703041 PMC1692717

[B128] Hilgetag C. C. Grant S. (2010). Cytoarchitectural differences are a key determinant of laminar projection origins in the visual cortex. Neuroimage 51, 1006–1017 10.1016/j.neuroimage.2010.03.006 20211270

[B129] Hilgetag C. C. Kaiser M. (2004). Clustered organization of cortical connectivity. Neuroinformatics 2, 353–360 10.1385/NI:2:3:353 15365196

[B130] Hilgetag C. C. Burns G. A. O'Neill M. A. Scannell J. W. Young M. P. (2000). Anatomical connectivity defines the organization of clusters of cortical areas in the macaque monkey and the cat. Philos. Trans. R. Soc. Lond. B Biol. Sci. 355, 91–110 10.1098/rstb.2000.0551 10703046 PMC1692723

[B131] Hill E. L. (2004). Executive dysfunction in autism. Trends Cogn. Sci. 8, 26–32 10.1016/j.tics.2003.11.003 14697400

[B132] Ho S. Clipstone N. Timmermann L. Northrop J. Graef I. Fiorentino D. (1996). The mechanism of action of cyclosporin A and FK506. Clin. Immunol. Immunopathol. 80, S40–S45 10.1006/clin.1996.0140 8811062

[B133] Hof P. R. Glezer I. I. Conde F. Flagg R. A. Rubin M. B. Nimchinsky E. A. (1999). Cellular distribution of the calcium-binding proteins parvalbumin, calbindin, and calretinin in the neocortex of mammals: phylogenetic and developmental patterns. J. Chem. Neuroanat. 16, 77–116 10.1016/S0891-0618(98)00065-9 10223310

[B134] Hogart A. Nagarajan R. P. Patzel K. A. Yasui D. H. Lasalle J. M. (2007). 15q11-13 GABAA receptor genes are normally biallelically expressed in brain yet are subject to epigenetic dysregulation in autism-spectrum disorders. Hum. Mol. Genet. 16, 691–703 10.1093/hmg/ddm014 17339270 PMC1934608

[B135] Holahan M. R. Honegger K. S. Tabatadze N. Routtenberg A. (2007). GAP-43 gene expression regulates information storage. Learn. Mem. 14, 407–415 10.1101/lm.581907 17554085 PMC1896091

[B136] Holahan M. Routtenberg A. (2008). The protein kinase C phosphorylation site on GAP-43 differentially regulates information storage. Hippocampus 18, 1099–1102 10.1002/hipo.20486 18727047 PMC2597099

[B137] Hornung J. P. De Tribolet N. (1994). Distribution of GABA-containing neurons in human frontal cortex: a quantitative immunocytochemical study. Anat. Embryol. (Berl.) 189, 139–145 10.1007/BF00185772 8010412

[B138] Howard C. V. Reed M. G. (1998). Unbiased Stereology, Three-dimensional Measurement in Microscopy. Oxford: BIOS Scientific Publishers Limited

[B139] Hsiao E. Y. McBride S. W. Chow J. Mazmanian S. K. Patterson P. H. (2012). Modeling an autism risk factor in mice leads to permanent immune dysregulation. Proc. Natl. Acad. Sci. U.S.A. 109, 12776–12781 10.1073/pnas.1202556109 22802640 PMC3411999

[B140] Hughes J. R. (2008). A review of recent reports on autism: 1000 studies published in 2007. Epilepsy Behav. 13, 425–437 10.1016/j.yebeh.2008.06.015 18627794

[B141] Hussman J. P. Chung R. Griswold A. Jaworski J. Salyakina D. Ma D. (2011). A noise-reduction GWAS analysis implicates altered regulation of neurite outgrowth and guidance in autism. Mol. Autism 2:1 10.1186/2040-2392-2-1 21247446 PMC3035032

[B142] Hutsler J. J. Zhang H. (2010). Increased dendritic spine densities on cortical projection neurons in autism spectrum disorders. Brain Res. 1309, 83–94 10.1016/j.brainres.2009.09.120 19896929

[B143] Hutsler J. J. Love T. Zhang H. (2007). Histological and magnetic resonance imaging assessment of cortical layering and thickness in autism spectrum disorders. Biol. Psychiatry 61, 449–457 10.1016/j.biopsych.2006.01.015 16580643

[B144] Hyde K. L. Samson F. Evans A. C. Mottron L. (2010). Neuroanatomical differences in brain areas implicated in perceptual and other core features of autism revealed by cortical thickness analysis and voxel-based morphometry. Hum. Brain Mapp. 31, 556–566 19790171 10.1002/hbm.20887 PMC6870833

[B145] Ito S. Stuphorn V. Brown J. W. Schall J. D. (2003). Performance monitoring by the anterior cingulate cortex during saccade countermanding. Science 302, 120–122 10.1126/science.1087847 14526085

[B146] Jacot-Descombes S. Uppal N. Wicinski B. Santos M. Schmeidler J. Giannakopoulos P. (2012). Decreased pyramidal neuron size in Brodmann areas 44 and 45 in patients with autism. Acta Neuropathol. 124, 67–79 10.1007/s00401-012-0976-6 22467063

[B147] Jiao Y. Chen R. Ke X. Y. Chu K. K. Lu Z. H. Herskovits E. H. (2010). Predictive models of autism spectrum disorder based on brain regional cortical thickness. Neuroimage 50, 589–599 10.1016/j.neuroimage.2009.12.047 20026220 PMC2823830

[B148] Johnston K. Levin H. M. Koval M. J. Everling S. (2007). Top-down control-signal dynamics in anterior cingulate and prefrontal cortex neurons following task switching. Neuron 53, 453–462 10.1016/j.neuron.2006.12.023 17270740

[B149] Jones E. G. (2009). Synchrony in the interconnected circuitry of the thalamus and cerebral cortex. Ann. N.Y. Acad. Sci. 1157, 10–23 10.1111/j.1749-6632.2009.04534.x 19351352

[B150] Jou R. J. Mateljevic N. Kaiser M. D. Sugrue D. R. Volkmar F. R. Pelphrey K. A. (2011). Structural neural phenotype of autism: preliminary evidence from a diffusion tensor imaging study using tract-based spatial statistics. Am. J. Neuroradiol. 32, 1607–1613 10.3174/ajnr.A2558 21799040 PMC7965377

[B151] Jou R. J. Minshew N. J. Keshavan M. S. Hardan A. Y. (2010). Cortical gyrification in autistic and asperger disorders: a preliminary magnetic resonance imaging study. J. Child Neurol. 25, 1462–1467 10.1177/0883073810368311 20413799 PMC3115701

[B152] Just M. A. Cherkassky V. L. Keller T. A. Minshew N. J. (2004). Cortical activation and synchronization during sentence comprehension in high-functioning autism: evidence of underconnectivity. Brain 127, 1811–1821 10.1093/brain/awh199 15215213

[B153] Just M. A. Cherkassky V. L. Keller T. A. Kana R. K. Minshew N. J. (2007). Functional and anatomical cortical underconnectivity in autism: evidence from an fMRI study of an executive function task and corpus callosum morphometry. Cereb. Cortex 17, 951–961 10.1093/cercor/bhl006 16772313 PMC4500121

[B154] Just M. A. Keller T. A. Malave V. L. Kana R. K. Varma S. (2012). Autism as a neural systems disorder: a theory of frontal-posterior underconnectivity. Neurosci. Biobehav. Rev. 36, 1292–1313 10.1016/j.neubiorev.2012.02.007 22353426 PMC3341852

[B155] Jyonouchi H. Sun S. Le H. (2001). Proinflammatory and regulatory cytokine production associated with innate and adaptive immune responses in children with autism spectrum disorders and developmental regression. J. Neuroimmunol. 120, 170–179 10.1016/S0165-5728(01)00421-0 11694332

[B156] Kalus P. Senitz D. (1996). Parvalbumin in the human anterior cingulate cortex: morphological heterogeneity of inhibitory interneurons. Brain Res. 729, 45–54 10.1016/0006-8993(96)00415-5 8874875

[B157] Kana R. K. Keller T. A. Cherkassky V. L. Minshew N. J. Just M. A. (2006a). Sentence comprehension in autism: thinking in pictures with decreased functional connectivity. Brain 129, 2484–2493 10.1093/brain/awl164 16835247 PMC4500127

[B158] Kana R. K. Keller T. A. Minshew N. J. Just M. A. (2006b). Inhibitory control in high-functioning autism: decreased activation and underconnectivity in inhibition networks. Biol. Psychiatry 62, 198–206 10.1016/j.biopsych.2006.08.004 17137558 PMC4492460

[B159] Kanazir S. Ruzdijic S. Vukosavic S. Ivkovic S. Milosevic A. Zecevic N. (1996). GAP-43 mRNA expression in early development of human nervous system. Brain Res. Mol. Brain Res. 38, 145–155 10.1016/0169-328X(96)00008-3 8737678

[B160] Kapfhammer J. P. Schwab M. E. (1994). Inverse patterns of myelination and GAP-43 expression in the adult CNS: neurite growth inhibitors as regulators of neuronal plasticity? J. Comp. Neurol. 340, 194–206 10.1002/cne.903400206 8201019

[B161] Kawaguchi Y. Kubota Y. (1997). GABAergic cell subtypes and their synaptic connections in rat frontal cortex. Cereb. Cortex 7, 476–486 10.1093/cercor/7.6.476 9276173

[B162] Keller T. A. Kana R. K. Just M. A. (2007). A developmental study of the structural integrity of white matter in autism. Neuroreport 18, 23–27 10.1097/01.wnr.0000239965.21685.99 17259855

[B163] Kennedy D. P. Redcay E. Courchesne E. (2006). Failing to deactivate: resting functional abnormalities in autism. Proc. Natl. Acad. Sci. U.S.A. 103, 8275–8280 10.1073/pnas.0600674103 16702548 PMC1472462

[B164] Khan S. Gramfort A. Shetty N. R. Kitzbichler M. G. Ganesan S. Moran J. M. (2013). Local and long-range functional connectivity is reduced in concert in autism spectrum disorders. Proc. Natl. Acad. Sci. U.S.A. 110, 3107–3112 10.1073/pnas.1214533110 23319621 PMC3581984

[B165] Kondo H. Tanaka K. Hashikawa T. Jones E. G. (1999). Neurochemical gradients along monkey sensory cortical pathways: calbindin-immunoreactive pyramidal neurons in layers II and III. Eur. J. Neurosci. 11, 4197–4203 10.1046/j.1460-9568.1999.00844.x 10594645

[B166] Koshino H. Kana R. K. Keller T. A. Cherkassky V. L. Minshew N. J. Just M. A. (2008). fMRI investigation of working memory for faces in autism: visual coding and underconnectivity with frontal areas. Cereb. Cortex 18, 289–300 10.1093/cercor/bhm054 17517680 PMC4500154

[B167] Kumar A. Sundaram S. K. Sivaswamy L. Behen M. E. Makki M. I. Ager J. (2009). Alterations in frontal lobe tracts and corpus callosum in young children with autism spectrum disorder. Cereb. Cortex 20, 2103–2113 10.1093/cercor/bhp278 20019145

[B168] Lai M. C. Lombardo M. V. Chakrabarti B. Sadek S. A. Pasco G. Wheelwright S. J. (2010). A shift to randomness of brain oscillations in people with autism. Biol. Psychiatry 68, 1092–1099 10.1016/j.biopsych.2010.06.027 20728872

[B169] Langen M. Leemans A. Johnston P. Ecker C. Daly E. Murphy C. M. (2012). Fronto-striatal circuitry and inhibitory control in autism: findings from diffusion tensor imaging tractography. Cortex 48, 183–193 10.1016/j.cortex.2011.05.018 21718979

[B170] Lawrence Y. A. Kemper T. L. Bauman M. L. Blatt G. J. (2010). Parvalbumin-, calbindin-, and calretinin-immunoreactive hippocampal interneuron density in autism. Acta Neurol. Scand. 121, 99–108 10.1111/j.1600-0404.2009.01234.x 19719810

[B171] Lee J. E. Bigler E. D. Alexander A. L. Lazar M. Dubray M. B. Chung M. K. (2007). Diffusion tensor imaging of white matter in the superior temporal gyrus and temporal stem in autism. Neurosci. Lett. 424, 127–132 10.1016/j.neulet.2007.07.042 17714869

[B172] Lepagnol-Bestel A. M. Maussion G. Boda B. Cardona A. Iwayama Y. Delezoide A. L. (2008). SLC25A12 expression is associated with neurite outgrowth and is upregulated in the prefrontal cortex of autistic subjects. Mol. Psychiatry 13, 385–397 10.1038/sj.mp.4002120 18180767

[B173] Letinic K. Kostovic I. (1998). Postnatal development of calcium-binding proteins calbindin and parvalbumin in human visual cortex. Cereb. Cortex 8, 660–669 10.1093/cercor/8.7.660 9823487

[B174] Levisohn P. M. (2007). The autism-epilepsy connection. Epilepsia 48 Suppl. 9, 33–35 10.1111/j.1528-1167.2007.01399.x 18047599

[B175] Levitt P. (2003). Structural and functional maturation of the developing primate brain. J. Pediatr. 143, S35–S45 10.1067/S0022-3476(03)00400-1 14597912

[B176] Lewis D. A. Morrison J. H. (1989). Noradrenergic innervation of monkey prefrontal cortex: a dopamine-á-hydoxylase immunohistochemical study. J. Comp. Neurol. 282, 317–330 10.1002/cne.902820302 2715385

[B177] Lewis D. A. Foote S. L. Goldstein M. Morrison J. H. (1988). The dopaminergic innervation of monkey prefrontal cortex: a tyrosine hydroxylase immunohistochemical study. Brain Res. 449, 225–243 10.1016/0006-8993(88)91040-2 2899447

[B178] London M. Hausser M. (2005). Dendritic computation. Annu. Rev. Neurosci. 28, 503–532 10.1146/annurev.neuro.28.061604.135703 16033324

[B179] Loveland K. A. Bachevalier J. Pearson D. A. Lane D. M. (2008). Fronto-limbic functioning in children and adolescents with and without autism. Neuropsychologia 46, 49–62 10.1016/j.neuropsychologia.2007.08.017 17936314 PMC2785231

[B180] Lowenstein P. R. Somogyi P. (1991). Synaptic organization of cortico-cortical connections from the primary visual cortex to the posteromedial lateral suprasylvian visual area in the cat. J. Comp. Neurol. 310, 253–266 10.1002/cne.903100209 1955584

[B181] Luna B. Minshew N. J. Garver K. E. Lazar N. A. Thulborn K. R. Eddy W. F. (2002). Neocortical system abnormalities in autism: an fMRI study of spatial working memory. Neurology 59, 834–840 10.1212/WNL.59.6.834 12297562

[B182] Ma D. Q. Whitehead P. L. Menold M. M. Martin E. R. Ashley-Koch A. E. Mei H. (2005). Identification of significant association and gene-gene interaction of GABA receptor subunit genes in autism. Am. J. Hum. Genet. 77, 377–388 10.1086/433195 16080114 PMC1226204

[B183] MacDonald A. W. III. Cohen J. D. Stenger V. A. Carter C. S. (2000). Dissociating the role of the dorsolateral prefrontal and anterior cingulate cortex in cognitive control. Science 288, 1835–1838 10.1126/science.288.5472.1835 10846167

[B184] Maestro S. Muratori F. Cavallaro M. C. Pei F. Stern D. Golse B. (2002). Attentional skills during the first 6 months of age in autism spectrum disorder. J. Am. Acad. Child Adolesc. Psychiatry 41, 1239–1245 10.1097/00004583-200210000-00014 12364846

[B185] Malkova N. V. Yu C. Z. Hsiao E. Y. Moore M. J. Patterson P. H. (2012). Maternal immune activation yields offspring displaying mouse versions of the three core symptoms of autism. Brain Behav. Immun. 26, 607–616 10.1016/j.bbi.2012.01.011 22310922 PMC3322300

[B186] Markram K. M. Markram H. (2010). The intense world theory - a unifying theory of the neurobiology of autism. Front. Hum. Neurosci. 4:224 10.3389/fnhum.2010.00224 21191475 PMC3010743

[B187] Maviel T. Durkin T. P. Menzaghi F. Bontempi B. (2004). Sites of neocortical reorganization critical for remote spatial memory. Science 305, 96–99 10.1126/science.1098180 15232109

[B188] Medalla M. Barbas H. (2006). Diversity of laminar connections linking periarcuate and lateral intraparietal areas depends on cortical structure. Eur. J. Neurosci. 23, 161–179 10.1111/j.1460-9568.2005.04522.x 16420426

[B189] Medalla M. Barbas H. (2009). Synapses with inhibitory neurons differentiate anterior cingulate from dorsolateral prefrontal pathways associated with cognitive control. Neuron 61, 609–620 10.1016/j.neuron.2009.01.006 19249280 PMC2804928

[B190] Medalla M. Barbas H. (2010). Anterior cingulate synapses in prefrontal areas 10 and 46 suggest differential influence in cognitive control. J. Neurosci. 30, 16068–16081 10.1523/JNEUROSCI.1773-10.2010 21123554 PMC3064955

[B191] Medalla M. Barbas H. (2012). The anterior cingulate cortex may enhance inhibition of lateral prefrontal cortex via m2 cholinergic receptors at dual synaptic sites. J. Neurosci. 32, 15611–15625 10.1523/JNEUROSCI.2339-12.2012 23115196 PMC3523186

[B192] Medalla M. Lera P. Feinberg M. Barbas H. (2007). Specificity in inhibitory systems associated with prefrontal pathways to temporal cortex in primates. Cereb. Cortex 17 Suppl. 1, i136–i150 10.1093/cercor/bhm068 17725996

[B193] Meskenaite V. (1997). Calretinin-immunoreactive local circuit neurons in area 17 of the cynomolgus monkey, *Macaca fascicularis*. J. Comp. Neurol. 379, 113–132 10.1002/(SICI)1096-9861(19970303)379:1<113::AID-CNE8>3.0.CO;2-7 9057116

[B194] Micheva K. D. Busse B. Weiler N. C. O'Rourke N. Smith S. J. (2010). Single-synapse analysis of a diverse synapse population: proteomic imaging methods and markers. Neuron 68, 639–653 10.1016/j.neuron.2010.09.024 21092855 PMC2995697

[B195a] Miguel-Hidalgo J. J. Overholser J. C. Meltzer H. Y. Stockmeier C. A. Rajkowska G. (2006). Reduced glial and neuronal packing density in the orbitofrontal cortex in alcohol dependence and its relationship with suicide and duration of alcohol dependence. Aging Clin. Exp. Res. 30, 1845–1855 10.1111/j.1530-0277.2006.00221.x 17067348 PMC2921167

[B195] Milosevic A. Kanazir S. Zecevic N. (1995). Immunocytochemical localization of growth-associated protein GAP-43 in early human development. Brain Res. Dev. Brain Res. 84, 282–286 10.1016/0165-3806(94)00187-5 7743648

[B196] Minshew N. J. Keller T. A. (2010). The nature of brain dysfunction in autism: functional brain imaging studies. Curr. Opin. Neurol. 23, 124–130 10.1097/WCO.0b013e32833782d4 20154614 PMC2975255

[B197] Minshew N. J. Williams D. L. (2007). The new neurobiology of autism: cortex, connectivity, and neuronal organization. Arch. Neurol. 64, 945–950 10.1001/archneur.64.7.945 17620483 PMC2597785

[B198] Morgan J. T. Chana G. Abramson I. Semendeferi K. Courchesne E. Everall I. P. (2012). Abnormal microglial-neuronal spatial organization in the dorsolateral prefrontal cortex in autism. Brain Res. 1456, 72–81 10.1016/j.brainres.2012.03.036 22516109

[B199] Morgan J. T. Chana G. Pardo C. A. Achim C. Semendeferi K. Buckwalter J. (2010). Microglial activation and increased microglial density observed in the dorsolateral prefrontal cortex in autism. Biol. Psychiatry 68, 368–376 10.1016/j.biopsych.2010.05.024 20674603

[B200] Mountcastle V. (1998). “The columnar organization of the neocortex,” in Perceptual Neuroscience: The Cerebral Cortex, ed Mountcastle V. (Cambridge: Harvard University Press), 165–203

[B201] Mountcastle V. B. (1997). The columnar organization of the neocortex. Brain 120(Pt 4), 701–722 10.1093/brain/120.4.701 9153131

[B202] Muller R. A. Shih P. Keehn B. Deyoe J. R. Leyden K. M. Shukla D. K. (2011). Underconnected, but how? A survey of functional connectivity MRI studies in autism spectrum disorders. Cereb. Cortex 21, 2233–2243 10.1093/cercor/bhq296 21378114 PMC3169656

[B203] Mundy P. (2003). Annotation: the neural basis of social impairments in autism: the role of the dorsal medial-frontal cortex and anterior cingulate system. J. Child Psychol. Psychiatry 44, 793–809 10.1111/1469-7610.00165 12959489

[B204] Murray E. A. Wise S. P. (2012). Why is there a special issue on perirhinal cortex in a journal called hippocampus? The perirhinal cortex in historical perspective. Hippocampus 22, 1941–1951 10.1002/hipo.22055 22987673 PMC3951742

[B205] Neve R. L. Finch E. A. Bird E. D. Benowitz L. I. (1988). Growth-associated protein GAP-43 is expressed selectively in associative regions of the adult human brain. Proc. Natl. Acad. Sci. U.S.A. 85, 3638–3642 10.1073/pnas.85.10.3638 3368468 PMC280269

[B206] Nguyen T. Lindner R. Tedeschi A. Forsberg K. Green A. Wuttke A. (2009). NFAT-3 is a transcriptional repressor of the growth-associated protein 43 during neuronal maturation. J. Biol. Chem. 284, 18816–18823 10.1074/jbc.M109.015719 19443652 PMC2707217

[B207] Nimchinsky E. A. Sabatini B. L. Svoboda K. (2002). Structure and function of dendritic spines. Annu. Rev. Physiol. 64, 313–353 10.1146/annurev.physiol.64.081501.160008 11826272

[B208] Norbury C. F. Brock J. Cragg L. Einav S. Griffiths H. Nation K. (2009). Eye-movement patterns are associated with communicative competence in autistic spectrum disorders. J. Child Psychol. Psychiatry 50, 834–842 10.1111/j.1469-7610.2009.02073.x 19298477

[B209] Oblak A. L. Gibbs T. T. Blatt G. J. (2010). Decreased GABA(B) receptors in the cingulate cortex and fusiform gyrus in autism. J. Neurochem. 114, 1414–1423 20557420 10.1111/j.1471-4159.2010.06858.x PMC2923229

[B210] Oblak A. L. Gibbs T. T. Blatt G. J. (2011a). Reduced GABAA receptors and benzodiazepine binding sites in the posterior cingulate cortex and fusiform gyrus in autism. Brain Res. 1380, 218–228 10.1016/j.brainres.2010.09.021 20858465 PMC3020259

[B211] Oblak A. L. Rosene D. L. Kemper T. L. Bauman M. L. Blatt G. J. (2011b). Altered posterior cingulate cortical cyctoarchitecture, but normal density of neurons and interneurons in the posterior cingulate cortex and fusiform gyrus in autism. Autism Res. 4, 200–211 10.1002/aur.188 21360830 PMC3110607

[B212] Oblak A. Gibbs T. T. Blatt G. J. (2009). Decreased GABAA receptors and benzodiazepine binding sites in the anterior cingulate cortex in autism. Autism Res. 2, 205–219 10.1002/aur.88 19650112 PMC2762426

[B213] Oishi T. Higo N. Umino Y. Matsuda K. Hayashi M. (1998). Development of GAP-43 mRNA in the macaque cerebral cortex. Brain Res. Dev. Brain Res. 109, 87–97 10.1016/S0165-3806(98)00067-4 9706394

[B214] Ostensen M. Khamashta M. Lockshin M. Parke A. Brucato A. Carp H. (2006). Anti-inflammatory and immunosuppressive drugs and reproduction. Arthritis Res.Ther. 8, 209 10.1186/ar1957 16712713 PMC1526635

[B215] Ozonoff S. Pennington B. F. Rogers S. J. (1991). Executive function deficits in high-functioning autistic individuals: relationship to theory of mind. J. Child Psychol. Psychiatry 32, 1081–1105 10.1111/j.1469-7610.1991.tb00351.x 1787138

[B216] Pardini M. Garaci F. G. Bonzano L. Roccatagliata L. Palmieri M. G. Pompili E. (2009). White matter reduced streamline coherence in young men with autism and mental retardation. Eur. J. Neurol. 16, 1185–1190 10.1111/j.1468-1331.2009.02699.x 19538216

[B217] Pardo C. A. Vargas D. L. Zimmerman A. W. (2005). Immunity, neuroglia and neuroinflammation in autism. Int. Rev. Psychiatry 17, 485–495 10.1080/02646830500381930 16401547

[B218] Patterson P. H. (2011). Maternal infection and immune involvement in autism. Trends Mol. Med. 17, 389–394 10.1016/j.molmed.2011.03.001 21482187 PMC3135697

[B219] Patterson P. H. (2012). Maternal infection and autism. Brain Behav. Immun. 26, 393 10.1016/j.bbi.2011.09.008 22001185

[B220] Paus T. (2001). Primate anterior cingulate cortex: where motor control, drive and cognition interface. Nat. Rev. Neurosci. 2, 417–424 10.1038/35077500 11389475

[B221] Paus T. Collins D. L. Evans A. C. Leonard G. Pike B. Zijdenbos A. (2001). Maturation of white matter in the human brain: a review of magnetic resonance studies. Brain Res. Bull. 54, 255–266 10.1016/S0361-9230(00)00434-2 11287130

[B222] Paus T. Zijdenbos A. Worsley K. Collins D. L. Blumenthal J. Giedd J. N. (1999). Structural maturation of neural pathways in children and adolescents: *in vivo* study. Science 283, 1908–1911 10.1126/science.283.5409.1908 10082463

[B223] Penzes P. Cahill M. E. Jones K. A. Vanleeuwen J. E. Woolfrey K. M. (2011). Dendritic spine pathology in neuropsychiatric disorders. Nat. Neurosci. 14, 285–293 10.1038/nn.2741 21346746 PMC3530413

[B224] Peters A. Sethares C. (1996). Myelinated axons and the pyramidal cell modules in monkey primary visual cortex. J. Comp. Neurol. 365, 232–255 10.1002/(SICI)1096-9861(19960205)365:2<232::AID-CNE3>3.0.CO;2-6 8822167

[B225] Peters A. Sethares C. (1997). The organization of double bouquet cells in monkey striate cortex. J. Neurocytol. 26, 779–797 10.1023/A:1018518515982 9482155

[B226] Peters A. Palay S. L. Webster H. D. (1991). The Fine Structure of the Nervous System. Neurons and Their Supporting Cells. New York, NY: Oxford University Press

[B227] Petrides M. Pandya D. N. (1988). Association fiber pathways to the frontal cortex from the superior temporal region in the rhesus monkey. J. Comp. Neurol. 273, 52–66 10.1002/cne.902730106 2463275

[B228] Petrides M. Pandya D. N. (2006). Efferent association pathways originating in the caudal prefrontal cortex in the macaque monkey. J.Comp Neurol. 498, 227–251 10.1002/cne.21048 16856142

[B229] Petrides M. Pandya D. N. (2007). Efferent association pathways from the rostral prefrontal cortex in the macaque monkey. J. Neurosci. 27, 11573–11586 10.1523/JNEUROSCI.2419-07.2007 17959800 PMC6673207

[B230] Pickett J. London E. (2005). The neuropathology of autism: a review. J. Neuropathol. Exp. Neurol. 64, 925–935 10.1097/01.jnen.0000186921.42592.6c 16254487

[B231] Raghanti M. A. Stimpson C. D. Marcinkiewicz J. L. Erwin J. M. Hof P. R. Sherwood C. C. (2008). Differences in cortical serotonergic innervation among humans, chimpanzees, and macaque monkeys: a comparative study. Cereb. Cortex 18, 584–597 10.1093/cercor/bhm089 17586605

[B232] Rakic P. (2002). Neurogenesis in adult primate neocortex: an evaluation of the evidence. Nat. Rev. Neurosci. 3, 65–71 10.1038/nrn700 11823806

[B233] Redcay E. Courchesne E. (2005). When is the brain enlarged in autism? A meta-analysis of all brain size reports. Biol. Psychiatry 58, 1–9 10.1016/j.biopsych.2005.03.026 15935993

[B234] Rempel-Clower N. L. Barbas H. (2000). The laminar pattern of connections between prefrontal and anterior temporal cortices in the rhesus monkey is related to cortical structure and function. Cereb. Cortex 10, 851–865 10.1093/cercor/10.9.851 10982746

[B235] Routtenberg A. Cantallops I. Zaffuto S. Serrano P. Namgung U. (2000). Enhanced learning after genetic overexpression of a brain growth protein. Proc. Natl. Acad. Sci. U.S.A. 97, 7657–7662 10.1073/pnas.97.13.7657 10861025 PMC16601

[B236] Rubenstein J. L. R. (2011). Development of the cerebral cortex: implications for neurodevelopmental disorders. J. Child Psychol. Psychiatry 52, 339–355 10.1111/j.1469-7610.2010.02307.x 20735793 PMC3429600

[B237] Rubenstein J. L. Merzenich M. M. (2003). Model of autism: increased ratio of excitation/inhibition in key neural systems. Genes Brain Behav. 2, 255–267 10.1034/j.1601-183X.2003.00037.x 14606691 PMC6748642

[B238] Sabbagh M. A. (2004). Understanding orbitofrontal contributions to theory-of-mind reasoning: implications for autism. Brain Cogn. 55, 209–219 10.1016/j.bandc.2003.04.002 15134854

[B239] Sairanen M. O'Leary O. F. Knuuttila J. E. Castren E. (2007). Chronic antidepressant treatment selectively increases expression of plasticity-related proteins in the hippocampus and medial prefrontal cortex of the rat. Neuroscience 144, 368–374 10.1016/j.neuroscience.2006.08.069 17049169

[B240] Samaco R. C. Hogart A. Lasalle J. M. (2005). Epigenetic overlap in autism-spectrum neurodevelopmental disorders: MECP2 deficiency causes reduced expression of UBE3A and GABRB3. Hum. Mol. Genet. 14, 483–492 10.1093/hmg/ddi045 15615769 PMC1224722

[B241] Santos M. Uppal N. Butti C. Wicinski B. Schmeidler J. Giannakopoulos P. (2011). Von Economo neurons in autism: a stereologic study of the frontoinsular cortex in children. Brain Res. 1380, 206–217 10.1016/j.brainres.2010.08.067 20801106

[B242] Schall J. D. (2001). Neural basis of deciding, choosing and acting. Nat. Rev. Neurosci. 2, 33–42 10.1038/35049054 11253357

[B243] Schipul S. Keller T. Just M. A. (2011). Inter-regional brain communication and its disturbance in autism. Front. Syst. Neurosci. 5:10 10.3389/fnsys.2011.00010 21390284 PMC3046360

[B244] Schmahmann J. D. Pandya D. N. (2006). Fiber Pathways of the Brain. New York, NY: Oxford University Press Inc 10.1093/acprof:oso/9780195104233.001.0001

[B245] Schmahmann J. D. Pandya D. N. Wang R. Dai G. D'Arceuil H. E. De Crespigny A. J. (2007). Association fibre pathways of the brain: parallel observations from diffusion spectrum imaging and autoradiography. Brain 130, 630–653 10.1093/brain/awl359 17293361

[B246] Schmitz C. Rezaie P. (2008). The neuropathology of autism: where do we stand? Neuropathol. Appl. Neurobiol. 34, 4–11 17971078 10.1111/j.1365-2990.2007.00872.x

[B247] Schumann C. M. Amaral D. G. (2006). Stereological analysis of amygdala neuron number in autism. J. Neurosci. 26, 7674–7679 10.1523/JNEUROSCI.1285-06.2006 16855095 PMC6674270

[B248] Schumann C. M. Nordahl C. W. (2011). Bridging the gap between MRI and postmortem research in autism. Brain Res. 1380, 175–186 10.1016/j.brainres.2010.09.061 20869352 PMC3050078

[B249] Schumann C. M. Bloss C. S. Barnes C. C. Wideman G. M. Carper R. A. Akshoomoff N. (2010). Longitudinal magnetic resonance imaging study of cortical development through early childhood in autism. J. Neurosci. 30, 4419–4427 10.1523/JNEUROSCI.5714-09.2010 20335478 PMC2859218

[B250] Schumann C. M. Hamstra J. Goodlin-Jones B. L. Lotspeich L. J. Kwon H. Buonocore M. H. (2004). The amygdala is enlarged in children but not adolescents with autism; the hippocampus is enlarged at all ages. J. Neurosci. 24, 6392–6401 10.1523/JNEUROSCI.1297-04.2004 15254095 PMC6729537

[B251] Selby L. Zhang C. Sun Q. Q. (2007). Major defects in neocortical GABAergic inhibitory circuits in mice lacking the fragile X mental retardation protein. Neurosci. Lett. 412, 227–232 10.1016/j.neulet.2006.11.062 17197085 PMC1839948

[B252a] Selemon L. D. Rajkowska G. Goldman-Rakic P. S. (1998). Elevated neuronal density in prefrontal area 46 in brains from schizophrenic patients: application of a three-dimensional, stereologic counting method. J. Comp. Neurol. 392, 402–412 10.1002/(SICI)1096-9861(19980316)392:3<402::AID-CNE9>3.0.CO;2-5 9511926

[B252] Seltzer B. Pandya D. N. (1989). Frontal lobe connections of the superior temporal sulcus in the rhesus monkey. J. Comp. Neurol. 281, 97–113 10.1002/cne.902810108 2925903

[B253] Shao Z. Burkhalter A. (1999). Role of GABAB receptor-mediated inhibition in reciprocal interareal pathways of rat visual cortex. J. Neurophysiol. 81, 1014–1024 10085329 10.1152/jn.1999.81.3.1014

[B254] Sherwood C. C. Raghanti M. A. Stimpson C. D. Spocter M. A. Uddin M. Boddy A. M. (2010). Inhibitory interneurons of the human prefrontal cortex display conserved evolution of the phenotype and related genes. Proc. Biol. Sci. 277, 1011–1020 10.1098/rspb.2009.1831 19955152 PMC2842764

[B255] Shukla D. K. Keehn B. Lincoln A. J. Muller R. A. (2010). White matter compromise of callosal and subcortical fiber tracts in children with autism spectrum disorder: a diffusion tensor imaging study. J. Am. Acad. Child Adolesc. Psychiatry 49, 1269–1278, 1278 e1261-1262. 21093776 10.1016/j.jaac.2010.08.018 PMC3346956

[B256] Shukla D. K. Keehn B. Muller R. A. (2011a). Tract-specific analyses of diffusion tensor imaging show widespread white matter compromise in autism spectrum disorder. J. Child Psychol. Psychiatry 52, 286–295 10.1111/j.1469-7610.2010.02342.x 21073464 PMC4547854

[B257] Shukla D. K. Keehn B. Smylie D. M. Muller R. A. (2011b). Microstructural abnormalities of short-distance white matter tracts in autism spectrum disorder. Neuropsychologia 49, 1378–1382 10.1016/j.neuropsychologia.2011.02.022 21333661 PMC3482113

[B258] Shulha H. P. Cheung I. Whittle C. Wang J. Virgil D. Lin C. L. (2012). Epigenetic signatures of autism: trimethylated H3K4 landscapes in prefrontal neurons. Arch. Gen. Psychiatry 69, 314–324 10.1001/archgenpsychiatry.2011.151 22065254

[B259] Simms M. L. Kemper T. L. Timbie C. M. Bauman M. L. Blatt G. J. (2009). The anterior cingulate cortex in autism: heterogeneity of qualitative and quantitative cytoarchitectonic features suggests possible subgroups. Acta Neuropathol. 118, 673–684 10.1007/s00401-009-0568-2 19590881

[B260] Simpson K. L. Weaver K. J. de Villers-Sidani E. Lu J. Y. Cai Z. Pang Y. (2011). Perinatal antidepressant exposure alters cortical network function in rodents. Proc. Natl. Acad. Sci. U.S.A. 108, 18465–18470 10.1073/pnas.1109353108 22025710 PMC3215047

[B261] Smith S. E. Li J. Garbett K. Mirnics K. Patterson P. H. (2007). Maternal immune activation alters fetal brain development through interleukin-6. J. Neurosci. 27, 10695–10702 10.1523/JNEUROSCI.2178-07.2007 17913903 PMC2387067

[B262] Somogyi P. Tamas G. Lujan R. Buhl E. H. (1998). Salient features of synaptic organisation in the cerebral cortex. Brain Res. Brain Res. Rev. 26, 113–135 10.1016/S0165-0173(97)00061-1 9651498

[B263] Sparks B. F. Friedman S. D. Shaw D. W. Aylward E. H. Echelard D. Artru A. A. (2002). Brain structural abnormalities in young children with autism spectrum disorder. Neurology 59, 184–192 10.1212/WNL.59.2.184 12136055

[B264] Srivastava D. P. Woolfrey K. M. Jones K. A. Anderson C. T. Smith K. R. Russell T. A. (2012). An autism-associated variant of Epac2 reveals a role for Ras/Epac2 signaling in controlling basal dendrite maintenance in mice. PLoS Biol. 10:e1001350 10.1371/journal.pbio.1001350 22745599 PMC3383751

[B265a] Stark A. K. Uylings H. B. Sanz-Arigita E. Pakkenberg B. (2004). Glial cell loss in the anterior cingulate cortex, a subregion of the prefrontal cortex, in subjects with schizophrenia. Am. J. Psychiatry 161, 882–888 10.1176/appi.ajp.161.5.882 15121654

[B265] Steele S. D. Minshew N. J. Luna B. Sweeney J. A. (2007). Spatial working memory deficits in autism. J. Autism Dev. Disord. 37, 605–612 10.1007/s10803-006-0202-2 16909311

[B266] Sundaram S. K. Kumar A. Makki M. I. Behen M. E. Chugani H. T. Chugani D. C. (2008). Diffusion tensor imaging of frontal lobe in autism spectrum disorder. Cereb. Cortex 18, 2659–2665 10.1093/cercor/bhn031 18359780 PMC2567426

[B267] Suzuki K. Sugihara G. Ouchi Y. Nakamura K. Futatsubashi M. Takebayashi K. (2013). Microglial activation in young adults with autism spectrum disorder. JAMA Psychiatry 70, 49–58 10.1001/jamapsychiatry.2013.272 23404112

[B268] Suzuki Y. Matsuzawa H. Kwee I. L. Nakada T. (2003). Absolute eigenvalue diffusion tensor analysis for human brain maturation. NMR Biomed. 16, 257–260 10.1002/nbm.848 14648885

[B269] Szatmari P. Paterson A. D. Zwaigenbaum L. Roberts W. Brian J. Liu X. Q. (2007). Mapping autism risk loci using genetic linkage and chromosomal rearrangements. Nat. Genet. 39, 319–328 10.1038/ng1985 17322880 PMC4867008

[B270] Tabuchi K. Blundell J. Etherton M. R. Hammer R. E. Liu X. Powell C. M. (2007). A neuroligin-3 mutation implicated in autism increases inhibitory synaptic transmission in mice. Science 318, 71–76 10.1126/science.1146221 17823315 PMC3235367

[B271] Tamura R. Kitamura H. Endo T. Hasegawa N. Someya T. (2010). Reduced thalamic volume observed across different subgroups of autism spectrum disorders. Psychiatry Res. 184, 186–188 10.1016/j.pscychresns.2010.07.001 20850279

[B272] Tanji J. Hoshi E. (2008). Role of the lateral prefrontal cortex in executive behavioral control. Physiol. Rev. 88, 37–57 10.1152/physrev.00014.2007 18195082

[B273] Thakkar K. N. Polli F. E. Joseph R. M. Tuch D. S. Hadjikhani N. Barton J. J. (2008). Response monitoring, repetitive behaviour and anterior cingulate abnormalities in autism spectrum disorders (ASD). Brain 131, 2464–2478 10.1093/brain/awn099 18550622 PMC2525446

[B274] Thatcher R. W. North D. M. Neubrander J. Biver C. J. Cutler S. Defina P. (2009). Autism and EEG phase reset: deficient GABA mediated inhibition in thalamo-cortical circuits. Dev. Neuropsychol. 34, 780–800 10.1080/87565640903265178 20183733

[B275] Thomson A. M. Deuchars J. (1997). Synaptic interactions in neocortical local circuits: dual intracellular recordings *in vitro*. Cereb. Cortex 7, 510–522 10.1093/cercor/7.6.510 9276176

[B276] Tommerdahl M. Tannan V. Holden J. K. Baranek G. T. (2008). Absence of stimulus-driven synchronization effects on sensory perception in autism: evidence for local underconnectivity? Behav. Brain Funct. 4, 19 10.1186/1744-9081-4-19 18435849 PMC2374789

[B277] Trikalinos T. A. Karvouni A. Zintzaras E. Ylisaukko-Oja T. Peltonen L. Jarvela I. (2006). A heterogeneity-based genome search meta-analysis for autism-spectrum disorders. Mol. Psychiatry 11, 29–36 10.1038/sj.mp.4001750 16189507

[B278] Tsatsanis K. D. Rourke B. P. Klin A. Volkmar F. R. Cicchetti D. Schultz R. T. (2003). Reduced thalamic volume in high-functioning individuals with autism. Biol. Psychiatry 53, 121–129 10.1016/S0006-3223(02)01530-5 12547467

[B279] van Kooten I. A. Palmen S. J. von Cappeln P. Steinbusch H. W. Korr H. Heinsen H. (2008). Neurons in the fusiform gyrus are fewer and smaller in autism. Brain 131, 987–999 10.1093/brain/awn033 18332073

[B280] Vargas D. L. Nascimbene C. Krishnan C. Zimmerman A. W. Pardo C. A. (2005). Neuroglial activation and neuroinflammation in the brain of patients with autism. Ann. Neurol. 57, 67–81 10.1002/ana.20315 15546155

[B281] Vlamings P. H. J. M. Jonkman L. M. Hoeksma M. R. van Engeland H. Kemner C. (2008). Reduced error monitoring in children with autism spectrum disorder: an ERP study. Eur. J. Neurosci. 28, 399–406 10.1111/j.1460-9568.2008.06336.x 18702711

[B282] Voineagu I. Wang X. C. Johnston P. Lowe J. K. Tian Y. Horvath S. (2011). Transcriptomic analysis of autistic brain reveals convergent molecular pathology. Nature 474, 380–384 10.1038/nature10110 21614001 PMC3607626

[B283] Von Bonin G. (1950). Essay on the Cerebral Cortex. Springfield, IL: Thomas, C.C

[B284a] Von Economo C. (2009). Cellular structure of the human cerebral cortex. Basel: Karger

[B284] Wang X. J. Tegner J. Constantinidis C. Goldman-Rakic P. S. (2004). Division of labor among distinct subtypes of inhibitory neurons in a cortical microcircuit of working memory. Proc. Natl. Acad. Sci. U.S.A. 101, 1368–1373 10.1073/pnas.0305337101 14742867 PMC337059

[B285] Wass S. (2011). Distortions and disconnections: disrupted brain connectivity in autism. Brain Cogn. 75, 18–28 10.1016/j.bandc.2010.10.005 21055864

[B286] Wegiel J. Kuchna I. Nowicki K. Imaki H. Marchi E. Ma S. Y. (2010). The neuropathology of autism: defects of neurogenesis and neuronal migration, and dysplastic changes. Acta Neuropathol. 119, 755–770 10.1007/s00401-010-0655-4 20198484 PMC2869041

[B287] Weidenheim K. M. Goodman L. Dickson D. W. Gillberg C. Rastam M. Rapin I. (2001). Etiology and pathophysiology of autistic behavior: clues from two cases with an unusual variant of neuroaxonal dystrophy. J. Child Neurol. 16, 809–819 10.1177/08830738010160110601 11732766

[B288] Weiss L. A. Arking D. E. Daly M. J. Chakravarti A. Brune C. W. West K. (2009). A genome-wide linkage and association scan reveals novel loci for autism. Nature 461, 802–808 10.1038/nature08490 19812673 PMC2772655

[B289] West M. J. Slomianka L. Gundersen H. J. G. (1991). Unbiased stereological estimation of the total number of neurons in the subdivisions of the rat hippocampus using the optical fractionator. Anat. Rec. 231, 482–497 10.1002/ar.1092310411 1793176

[B290] White E. L. (1989). Cortical Circuits. Synaptic Organization of the Cerebral Cortex. Structure, Function and Theory. Boston, MA: Birkhäuser 10.1007/978-1-4684-8721-3

[B291] White J. A. Banks M. I. Pearce R. A. Kopell N. J. (2000). Networks of interneurons with fast and slow gamma-aminobutyric acid type A (GABAA) kinetics provide substrate for mixed gamma-theta rhythm. Proc. Natl. Acad. Sci. U.S.A. 97, 8128–8133 10.1073/pnas.100124097 10869419 PMC16681

[B292] Whittington M. A. Cunningham M. O. Lebeau F. E. Racca C. Traub R. D. (2011). Multiple origins of the cortical gamma rhythm. Dev. Neurobiol. 71, 92–106 10.1002/dneu.20814 21154913

[B293] Whittington M. A. Traub R. D. Kopell N. Ermentrout B. Buhl E. H. (2000). Inhibition-based rhythms: experimental and mathematical observations on network dynamics. Int. J. Psychophysiol. 38, 315–336 10.1016/S0167-8760(00)00173-2 11102670

[B294] Wilson T. W. Rojas D. C. Reite M. L. Teale P. D. Rogers S. J. (2007). Children and adolescents with autism exhibit reduced MEG steady-state gamma responses. Biol. Psychiatry 62, 192–197 10.1016/j.biopsych.2006.07.002 16950225 PMC2692734

[B295] Wong K. L. Murakami K. Routtenberg A. (1989). Dietary cis-fatty acids that increase protein F1 phosphorylation enhance spatial memory. Brain Res. 505, 302–305 10.1016/0006-8993(89)91456-X 2598047

[B296] Yakovlev P. I. Lecours A. R. (1967). “The myelogenetic cycles of regional maturation of the brain,” in Regional Development of the Brain in Early Life, ed Minowski A. (Oxford and Edinburgh: Blackwell Scientific Publications), 3–70

[B297] Yan X. X. Cao Q. L. Luo X. G. Garey L. J. (1997). Prenatal development of calbindin D-28K in human visual cortex. Cereb. Cortex 7, 57–62 10.1093/cercor/7.1.57 9023432

[B298] Yip J. Soghomonian J. J. Blatt G. J. (2008). Increased GAD67 mRNA expression in cerebellar interneurons in autism: implications for Purkinje cell dysfunction. J. Neurosci. Res. 86, 525–530 10.1002/jnr.21520 17918742

[B299] Yizhar O. Fenno L. E. Prigge M. Schneider F. Davidson T. J. O'Shea D. J. (2011). Neocortical excitation/inhibition balance in information processing and social dysfunction. Nature 477, 171–178 10.1038/nature10360 21796121 PMC4155501

[B300] Yoshida T. Mishina M. (2005). Distinct roles of calcineurin-nuclear factor of activated T-cells and protein kinase A-cAMP response element-binding protein signaling in presynaptic differentiation. J. Neurosci. 25, 3067–3079 10.1523/JNEUROSCI.3738-04.2005 15788763 PMC6725083

[B301] Zaccaria K. J. Lagace D. C. Eisch A. J. McCasland J. S. (2010). Resistance to change and vulnerability to stress: autistic-like features of GAP43-deficient mice. Genes Brain Behav. 9, 985–996 10.1111/j.1601-183X.2010.00638.x 20707874 PMC2975747

[B302] Zikopoulos B. Barbas H. (2006). Prefrontal projections to the thalamic reticular nucleus form a unique circuit for attentional mechanisms. J. Neurosci. 26, 7348–7361 10.1523/JNEUROSCI.5511-05.2006 16837581 PMC6674204

[B303] Zikopoulos B. Barbas H. (2007). Parallel driving and modulatory pathways link the prefrontal cortex and thalamus. PLoS ONE 2:e848 10.1371/journal.pone.0000848 17786219 PMC1952177

[B304] Zikopoulos B. Barbas H. (2010). Changes in prefrontal axons may disrupt the network in autism. J. Neurosci. 30, 14595–14609 10.1523/JNEUROSCI.2257-10.2010 21048117 PMC3073590

[B305] Zikopoulos B. Barbas H. (2012). Pathways for emotions and attention converge on the thalamic reticular nucleus in primates. J. Neurosci. 32, 5338–5350 10.1523/JNEUROSCI.4793-11.2012 22496579 PMC3342673

[B306] Zikopoulos B. Hoistad M. Barbas H. (2008). “Differential projections and synaptic interactions of posterior orbitofrontal and anterior cingulate cortices with the amygdala,” in Program No.35.5.2008 Neuroscience 2008 Abstracts, Vol. 34. (Washington, DC: Society for Neuroscience [Online]).

